# The importance of claudin-7 palmitoylation on membrane subdomain localization and metastasis-promoting activities

**DOI:** 10.1186/s12964-015-0105-y

**Published:** 2015-06-09

**Authors:** Sarah Heiler, Wei Mu, Margot Zöller, Florian Thuma

**Affiliations:** Department of Tumor Cell Biology, University Hospital of Surgery, Im Neuenheimer Feld 365, 69120 Heidelberg, Germany

**Keywords:** EpCAM, claudin7, Palmitoylation, Cleavage, Membrane microdomains

## Abstract

**Background:**

Claudin-7 (cld7), a tight junction (TJ) component, is also found basolaterally and in the cytoplasm. Basolaterally located cld7 is enriched in glycolipid-enriched membrane domains (GEM), where it associates with EpCAM (EpC). The conditions driving cld7 out of TJ into GEM, which is associated with a striking change in function, were not defined. Thus, we asked whether cld7 serines or palmitoylation affect cld7 location and protein, particularly EpCAM, associations.

**Results:**

HEK cells were transfected with EpCAM and wild type cld7 or cld7, where serine phopsphorylation or the palmitoylation sites (AA184, AA186) (cld7^mPalm^) were mutated. Exchange of individual serine phosphorylation sites did not significantly affect the GEM localization and the EpCAM association. Instead, cld7^mPalm^ was poorly recruited into GEM. This has consequences on migration and invasiveness as palmitoylated cld7 facilitates integrin and EpCAM recruitment, associates with cytoskeletal linker proteins and cooperates with MMP14, CD147 and TACE, which support motility, matrix degradation and EpCAM cleavage. On the other hand, only cld7^mPalm^ associates with TJ proteins.

**Conclusion:**

Cld7 palmitoylation prohibits TJ integration and fosters GEM recruitment. Via associated molecules, palmitoylated cld7 supports motility and invasion.

**Electronic supplementary material:**

The online version of this article (doi:10.1186/s12964-015-0105-y) contains supplementary material, which is available to authorized users.

## Introduction

Claudins (cld), a family of closely related four-pass molecules, are essential components of tight junctions (TJ) in the apical region of epithelial cells. TJ, which include JAM and occludins, regulate paracellular permeability and epithelial cell polarity [1-4]. As tumor cell dissemination requires loss of cell-cell adhesion, it was expected that claudins be downregulated in cancer. This however was not consistently observed [3,5-7]. Fittingly, claudins, most frequently cld7, also are recovered on the lateral or basal surface [6,8-10]. However, functional activities of claudins out of TJ are poorly defined [5].

Claudins have two palmitoylation sites towards the inner membrane site of the second and forth transmembrane domain [11,12]. As described for G-protein coupled receptors and tetraspanins [13-15], palmitoylation promotes the recruitment into glycolipid-enriched membrane microdomains (GEM) [16,17], which gather signal transduction [16,18,19] and cytoskeleton anchoring molecules [20]. Palmitoylation also promotes internalization and degradation or integration into multivesicular bodies and exosome delivery [21-24]. Claudins also have several phosphorylation sites [25,26]. Cld phosphorylation can prohibit integration into TJ [27], but distinct claudins may respond differently [28,29].

Claudins interact between themselves, additional TJ components and TJ-independent membrane molecules. CD9 and CD81 associate with cld1, the latter being essential for hepatitis C virus infection [30-32]. The interaction of EphrinB1 and EphrinA2 with cld4 affects barrier functions and increases leakiness [33,34]. We described that cld7 directly associated with EpCAM (EpC) in the transmembrane region [35]. EpC-cld7 co-localization is seen in colon, pancreatic and anaplastic thyroid carcinoma [36,37]. Pursuing in a rat pancreatic adenocarcinoma the impact of cld7 on EpC activity and vice versa revealed that cld7 recruits EpC into GEM, where both molecules associate with the tetraspanin Tspan8 and additional transmembrane molecules. These latter associations likely are not based on direct protein-protein interactions [36,37]. A cld7^kd^ and an EpC^kd^ revealed a striking reduction in metastasis formation, where cld7 supports tumorigenic features of EpC by provoking EpC cleavage and thereby the cotranscription factor activity of its intracellular domain (EpIC) [38,39]. Cld7 also associates with EpC in hepatocyte progenitors [40]. In congenital tufting enteropathy, characterized by a deletion of EpC exon 4, the loss of co-localization with cld7 is lethal [41]. Notably, an EpC^ko^ is associated with intestine destruction-promoted death within one week after birth, due to the missing association of EpC with cld7, which is accompanied by reduced cld7 expression [42]. The pathology resembles that of cld7^ko^ mice, which also die within 1 week after birth, due to altered barrier functions and inflammation-promoted gut destruction. The authors discuss the importance of a missing association with integrins and a striking up-regulation of MMP3 [43].

These findings strengthening our hypothesis on TJ-independent functions and cooperations of cld7 under (patho)physiological conditions [44-47], we aimed to elaborate the molecular mechanism prohibiting cld7 integration into TJ. As a model we choose HEK cells, which express cld7 at a very low level. HEK cells were transfected with serine- and palmitoylation-site-mutated cld7. We focused on cld7 palmitoylation, as palmitoylation is of critical importance for GEM localization, complex formation and recruitment of cytoskeletal linker and cytosolic signaling molecules [48-51]. To control for activities that depend on the cooperation with EpC, HEK cells were concomitantly transfected with EpC cDNA or a point mutated EpC cDNA that does not associate with cld7. Cld7 palmitoylation prohibits integration into the TJ protein complex. Instead, palmitoylated cld7 is preferentially recruited into GEM, where it associates with integrins and cytoskeletal linker proteins, which promote motility. Motility is additionally strengthened by cleavage of palmitoylated cld7-associated EpC, the co-transcription factor EpIC (EpC intracellular domain) supporting mesenchymal gene expression. Finally, palmitoylated cld7 associates with proteases, which strongly affects invasiveness.

## Results

The activity of cld7 outside of TJ is still disputed. We described recovery of cld7 in GEM, where it associates with EpC, tetraspanins and integrins, which supports motility and possibly the epithelial mesenchymal transition (36,52). The molecular basis for driving cld7 out of TJ into GEM as well as the GEM-location-dependent associations remained to be explored. We approached these questions by transfecting HEK cells with cld7, where serine phosphorylation sites (mS) and the palmitoylation site at the C-terminal cytoplasmic tail (mPalm) were mutated. The impact particularly on associating EpC was controlled by co-transfecting HEK cells with EpC, exchanging AA279 and AA282 by isoleucin (mAG), which prohibits cld7 binding (35,36).

### Claudin7 palmitoylation affects membrane subdomain localization and the association with EpC

We first evaluated the impact of serine phosphorylation on cld7 recruitment into GEM and on the association with EpC. HEK-EpC cells were transfected with seven different serine phosphorylation site point-mutated cld7 constructs (Additional file 1). WB showed comparable cld7 expression levels in non-mutated/mutated cld7 transfected HEK-EpC cells (Additional file 2a). The exchange of cld7 serines did not affect co-immunoprecipitation of EpC with cld7 (Additional file 2b). WB of sucrose density gradient fractions revealed partial recovery of cld7 in GEM. In the absence of cld7, EpC is mostly recovered in heavy fractions, but becomes partially recruited into light fractions in the presence of cld7. The distribution of EpC in light sucrose density fractions corresponded to the distribution of cld7. Recovery of EpC in GEM fractions varied between 55%-72% and of cld7 between 57%-73%. Thus, single serine mutations did not strongly affect or abolish GEM localization of cld7 and the recruitment of EpC into GEM (Additional file 2c). Co-immunoprecipitation confirmed that anti-EpC precipitates cld7 and anti-cld7 precipitates EpC. Irrespective of cld7 serine mutations, ~60%-70% of EpC-cld7 complexes are recovered in GEM. This is demonstrated for cld7^mS33^, cld7^mS69^, cld7^mS87^ and cld7^mS204^ (Additional file 2d), and also accounted for cld7^mS172^, cld7^mS206^ and cld7^mS207^ (data not shown).

We concluded that cld7 is preferentially located in GEM and recruits EpC into GEM. Single serine phosphorylation of cld7 does not considerably contribute to the EpC-cld7 association or to the membrane distribution of cld7 and the EpC-cld7 complex.

**Figure 1:**
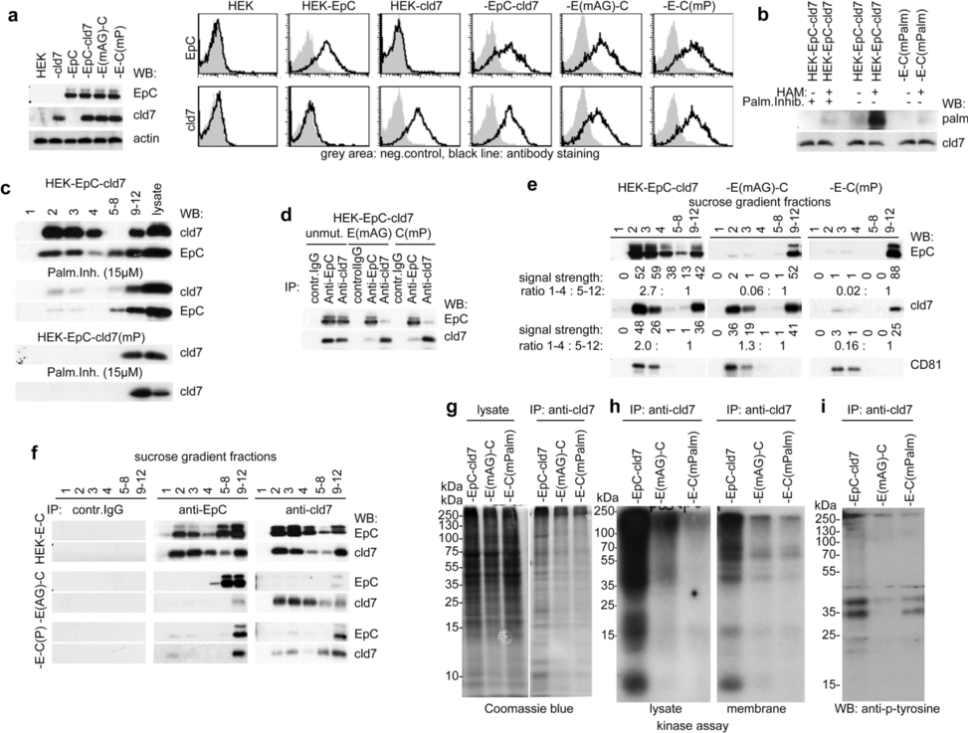
*Claudin-7 palmitoylation: Impact on membrane subdomain localization and protein association* **(a-e)** HEK cells were transfected with EpC and/or wt cld7 or EpC with a mutation of (mAG) in the transmembrane region or with cld7, with a palmitoylation site mutation. **(a)** WB of non-transfected and transfected HEK with anti-EpC, anti-cld7 and anti-actin (loading control); an example of flow cytometry analysis of EpC and cld7 in transfected HEK cells: single fluorescence overlays of negative controls and anti-EpC or anti-cld7 staining; **(b)** HEK-EpC-cld7 and HEK-EpC^mAG^-cld7 were lysed in the presence of N-ethylmaleimide to irreversibly block unmodified thiol groups. After incubation with HAM buffer for unmasking palmitoylated cysteine thiol groups, samples where incubated in biotin-BMCC for selective labeling palmitoylated cysteines. Samples were blotted with streptavidin-HRP and after stripping with anti-cld7. **(c)** WB of sucrose density fractions of untreated and 2-BP-treated HEK-EpC-cld7 and HEK-EpC-cld7^mPalm^ with anti-cld7 and anti-EpC; **(d)** IP of HEK-EpC-cld7, HEK-EpC^mAG^-cld7 and HEK-EpC-cld7^mPalm^ lysates with control IgG, anti-EpC and anti-cld7 and WB with anti-EpC and anti-cld7; **(e)** WB of non-mutated and mutated EpC and cld7 in light and heavy density fractions after density gradient centrifugation; constitutively GEM-located CD81 served as control; relative protein band intensity and the protein ratio in light to heavy fractions is indicated; **(f)** immunoprecipitation with control IgG, anti-EpC and anti-cld7 of lysates of non-mutated and mutated EpC and cld7 in light and heavy density fractions and WB with anti-EpC and anti-cld7; **(g-i)** SDS-PAGE, Coomassie-blue staining, *in vitro* kinase assay of whole cell and membrane lysates and WB with anti-p-tyrosine of anti-cld7 immunoprecipitates of HEK-EpC-cld7, HEK-EpC^mAG^-cld7 and HEK-EpC-cld7^mPalm^ lysates. Co-transfection of HEK-EpC with cld7 shifts EpC into GEM. Instead, recovery of EpC^mAG^ in GEM and co-immunoprecipitation with cld7 is strikingly reduced. Palmitoylation site mutated cld7 is hardly recovered in GEM and only few, mostly non-palmitoylated molecules associate with cld7^mPalm^.

We proceeded evaluating the impact of cld7 palmitoylation, a modification known to support protein anchoring in GEM [16,17]. HEK-EpC cells were transfected with cld7 carrying a mutation of the palmitoylation site at the C-terminal tail (HEK-EpC-cld7^mPalm^). To control, in addition, whether only cld7-associated EpC becomes recruited into GEM, HEK-cld7 cells were transfected with EpC cDNA mutated at AA279 and AA282 (HEK-EpC^mAG^-cld7), which account for the EpC-cld7 association [36]. Flow cytometry and WB confirmed comparable EpC / EpC^mAG^ and cld7 / cld7^mPalm^ expression in transfected HEK cells (Figure 1a).

The efficacy of inhibiting cld7 palmitoylation by the exchange of cysteine AA184 and AA186 was controlled by immunoprecipitation and acyl-biotin exchange in HEK-EpC-cld7 and HEK-EpC-cld7^mPalm^ lysates in the absence or presence of the palmitoylation inhibitor 2-BP. No palmitoylation signal was detected in the presence of 2-BP, but a strong signal was seen in HEK-EpC-cld7 lysates. A very faint band remained in HEK-EpC-cld7^mPalm^. Stripping the gel and blotting with anti-cld7 confirmed equal loading. From there we conclude that mutating AA184 and AA186 sufficed to prevent palmitoylation and also that the C-terminal palmitoylation site mostly accounts for cld7 palmitoylation (Figure 1b). We next evaluated the impact of mutating the cld7 palmitoylation site on the recruitment into GEM and the association with EpC using the palmitoylation inhibitor (2-BP) [53]. WB of 2-BP-treated HEK-EpC-cld7 cells showed a striking redistribution of cld7 with >90% recovery in the heavy fraction. The same accounted for EpC. On the contrary, cld7 was not recovered in GEM and 2-BP treatment had no impact on the cld7 GEM distribution in HEK-EpC-cld7^mPalm^ (Figure 1c). Thus, a considerable part of cld7 is palmitoylated and palmitoylation accounts for GEM recruitment. The finding also strengthens our interpretation that cld7 recruits EpC towards GEM. We proceeded with HEK-EpC-cld7^mPalm^ and HEK-EpC^mAG^-cld7 cells, where selectively cld7 palmitoylation and the cld7-EpC association should be distorted.

Co-immunoprecipitation of EpC and cld7 was strikingly reduced in HEK-EpC^mAG^-cld7 and HEK-EpC-cld7^mPalm^ lysates (Figure 1d). In HEK-EpC^mAG^, EpC was recovered in heavy sucrose gradient fractions, but cld7 remained enriched in GEM. Instead, cld7^mPalm^ partly shifted towards the dense fractions. Enrichment of the constitutively GEM-located tetraspanin CD81 in fraction 2–5 was independent of cld7 palmitoylation (Figure 1e). Furthermore, EpC was not detected in light and was poorly recovered in heavy density fractions in anti-cld7 immunoprecipitates of HEK-EpC-cld7^mPalm^ lysates. In HEK-EpC^mAG^-cld7 lysates, anti-EpC weakly co-imunoprecipitated cld7 only in heavy density fractions, but cld7 was still recovered in light density fractions of anti-cld7 precipitates (Figure 1f). Furthermore, cld7^mPalm^ and EpC^mAG^ severely influenced the phosphorylation status of associated molecules. First to note, SDS-PAGE and Coomassie blue staining of anti-cld7 precipitates revealed a reduction of co-immunoprecipitating molecules in EpC^mAG^ and more pronounced cld7^mPalm^ lysates, indicating that EpC- and cld7-associating molecules are partly overlapping (Figure 1g). Notably, a kinase assay revealed phosphorylation of a considerable number of molecules co-immunoprecipitating with cld7 in HEK-EpC-cld7, but not in HEK-EpC^mAG^-cld7 or HEK-EpC-cld7^mPalm^ lysates. The finding was confirmed in a kinase assay with lysates of the membrane fraction. In addition, recovery of ~15 kDa and ~11 kDa phosphorylated proteins, which abundantly immunoprecipitated in whole cell lysates, was poor in the membrane fraction IP. This could be indicative for loosely attached cytosolic proteins (Figure 1h). These molecules and a ~60 kDa protein are exclusively serine or threonine phosphorylated as they were not recovered in an anti-p-tyrosine blot (Figure 1i). We conclude that mostly serine/threonine phosphorylated proteins associated exclusively with palmitoylated cld7.

MALDI-TOF analysis of lower molecular weight proteins of HEK-EpC-cld7, HEK-EpC^mAG^-cld7 and HEK-EpC-cld7^mPalm^ lysates, which co-immunoprecipitated with cld7, confirmed that a considerable number of proteins co-immunoprecipitated with cld7 only in HEK-EpC-cld7, but not in HEK-EpC^mAG^-cld7 and, more pronounced in HEK-EpC-cld7^mPalm^ lysates. Due to very mild lysis condition, many co-immunoprecipitating molecules may be loosely attached and be part of larger protein complexes. Notably, the majority of molecules co-immunoprecipitating with cld7 in HEK-EpC-cld7, HEK-EpC^mAG^-cld7 and HEK-EpC-cld7^mPalm^ lysates is engaged in metabolism and ion transport. Instead, an unexpectedly high number of molecules co-immunoprecipitating with cld7 only in HEK-EpC-cld7 lysates is engaged in vesicle transport (Additional file 3).

Briefly, (i) cld7 palmitoylation is essential for stabilization in GEM and for the association with EpC and other transmembrane and membrane-associated molecules; (ii) the association with palmitoylation-competent cld7 severely affects the activation state of co-immunoprecipitating molecules; (iii) molecules associated with vesicle traffic preferentially associated with palmitoylation-competent cld7, which strengthens our hypothesis of distinct functions of palmitoylated versus non-palmitoylated cld7. The selectivity of palmitoylated cld7 membrane subdomain localization and protein associations offered the possibility to control for palmitoylation-dependent cld7 activities.

### Cld7 palmitoylation interferes with cell-cell adhesion

Formation of TJ is one of the central activities of clds [54]. As cld7 is also recovered outside of TJ and palmitoylation strongly affects cld7 location [35,54,55], we first asked, whether palmitoylation directly affects cld7 integration into TJ.

**Figure 2:**
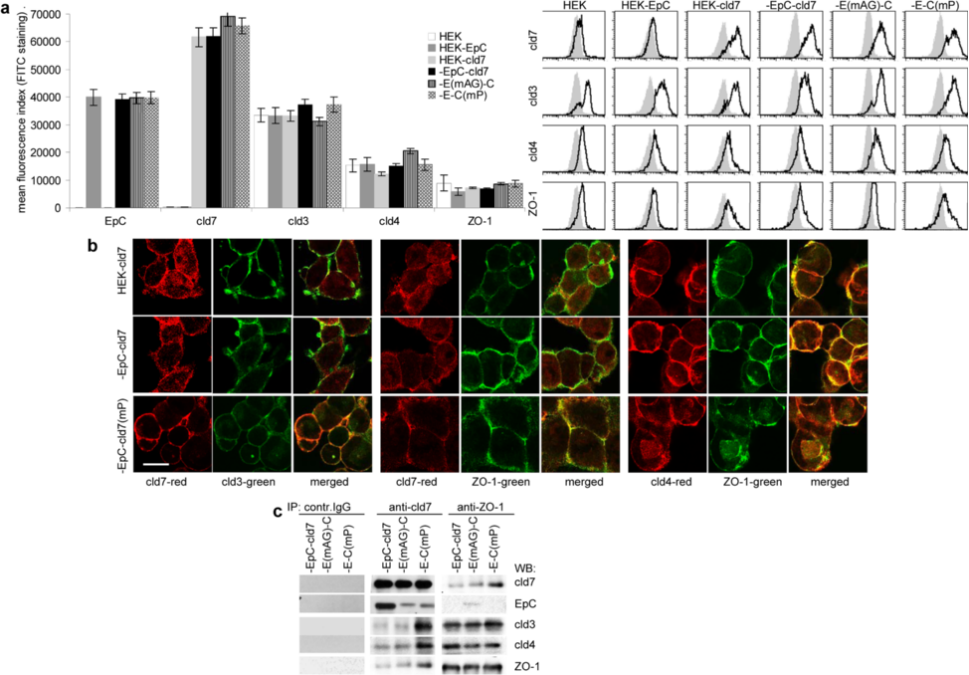
*Claudin7 palmitoylation and TJ formation* **(a)** Flow cytometry analysis of claudin and ZO-1 expression in HEK cells; the mean fluorescence index (% stained cells x mean intensity of staining ± SD; three assays) and representative examples are shown; **(b)** colocalization of cld7 with cld3 and ZO-1 and of cld4 with ZO-1 (scale bar: 10 μm); **(c)** immunoprecipitation with control IgG, anti-cld7 and anti-ZO-1 and co-immunoprecipitation of cld7, EpC, cld3, cld4 and ZO-1 in HEK-EpC-cld7, HEK-EpC^mAG^-cld7 and HEK-EpC-cld7^mPalm^ lysates. Palmitoylated cld7 poorly colocalizes and co-immunoprecipitates with TJ proteins.

HEK cells express cld3 at a high, ZO-1 and cld4 at an intermediate and cld7 at a very low level (Figure 2a). Cld7 poorly colocalizes with cld3 or ZO-1. However, there is a tendency towards more pronounced colocalization of palmitoylation-deficient cld7 with cld3 and ZO-1. Distinct to cld7, cld4 readily colocalizes with ZO-1 (Figure 2b). Co-immunoprecipitation confirmed that only cld7^mPalm^, but not palmitoylation-competent cld7 associates with cld3, cld4 and ZO-1. Instead, anti-ZO-1 precipitates cld3 and cld4, but only cld7^mPalm^ (Figure 2c). Thus, only non-palmitoylated / palmitoylation-deficient cld7 may contribute to TJ formation.

**Figure 3:**
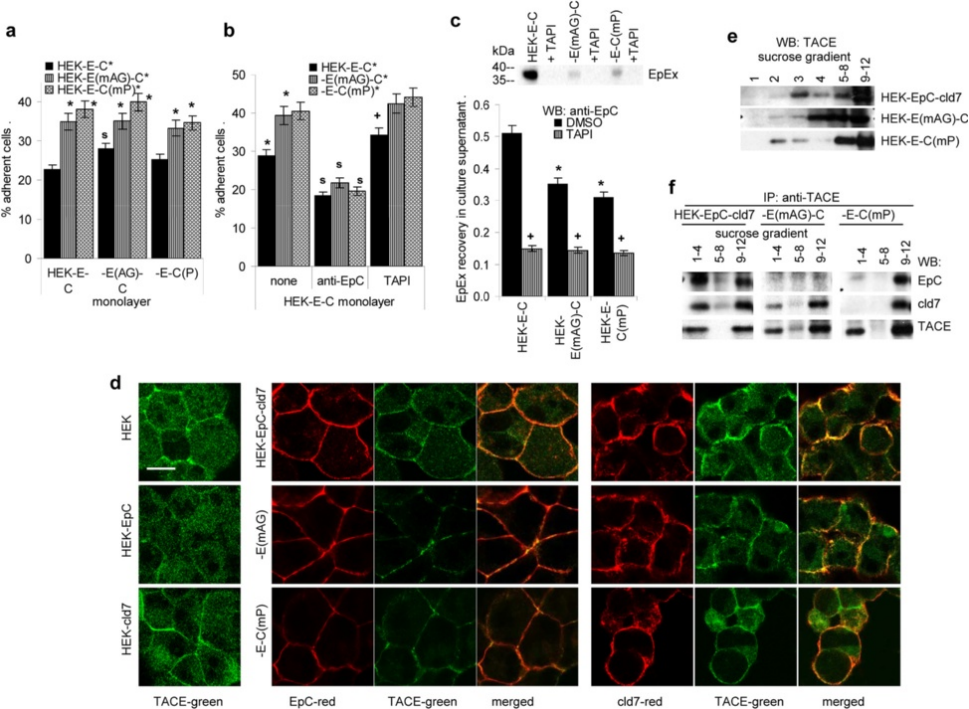
*Claudin7 palmitoylation and homophilic EpC cell-cell adhesion* **(a,b)** CFSE-labeled transfected HEK cells were seeded on a monolayer of HEK-EpC-cld7 cells with/without mutations of EpC or cld7. Where indicated, cells were cultured in the presence of anti-EpC (10 μg / ml) or TAPI (50 μM / ml). The percent of adherent cells (mean ± SD; triplicates) is shown; significant differences between HEK-EpC-cld7 and HEK-EpC^mAG^-cld7 or HEK-EpC-cld7^mPam^: *, significant differences by anti-EpC: s; significant differences in the presence of TAPI: +. **(c)** Supernatant of confluent PMA stimulated HEK-EpC-cld7, HEK-EpC^mAG^-cld7 and HEK-EpC-cld7^mPalm^ cells was collected after 48 h of culture in the absence of FCS. Where indicated cultures contained 50 μM / ml TAPI. Cell debris- and vesicle-depleted supernatant were adjusted to 100 μg / ml protein, were separated by SDS-PAGE and after transfer were blotted with anti-EpC or ELISA plates were coated with the concentrated supernatant. After blocking, EpEx was detected by anti-EpC binding. The OD at 595 nm (mean ± SD; triplicates) is shown; significant differences between HEK-EpC-cld7 and HEK-EpC^mAG^-cld7 or HEK-EpC-cld7^mPalm^: *, significant differences in the presence of TAPI: +. **(d)** Confocal microscopy of HEK, HEK-EpC, HEK-cld7, HEK-EpC-cld7, HEK-EpC^mAG^-cld7 and HEK-EpC-cld7^mPalm^ cells stained with anti-TACE and anti-EpC or anti-cld7. Green and red fluorescence and overlays of red and green fluorescence are shown (scale bar: 10 μm). **(e)** Lysates of cells as above were separated by sucrose gradient centrifugation and blotted with anti-TACE. **(f)** Lysates of the cells as above were precipitated with anti-TACE. After sucrose gradient centrifugation of the precipitates, SDS-PAGE and transfer, membranes were blotted with anti-EpC, anti-cld7 and anti-TACE. Palmitoylated cld7 interferes with homophilic EpC adhesion, which is partly due to the association of palmitoylated cld7 with TACE in GEM, which promotes EpEx cleavage.

Similar to cld7, EpC is a homophilic cell-cell adhesion molecule [56]. To control for a potential impact of the association with cld7, CFSE-labeled HEK-EpC-cld7, HEK-EpC^mAG^-cld7 and HEK-EpC-cld7^mPalm^ cells were seeded on a monolayer of HEK-EpC-cld7, HEK-EpC^mAG^-cld7 or HEK-EpC-cld7^mPalm^ cells. Slightly more HEK-EpC^mAG^-cld7 and HEK-EpC-cld7^mPalm^ than HEK-EpC-cld7 cells adhered to the HEK-EpC-cld7 monolayer (Figure 3a). Increased cell-cell adhesion of HEK-EpC^mAG^-cld7 was due to EpC binding, as it was inhibited by anti-EpC (Figure 3b). This finding supports the interpretation that GEM-located cld7 also interferes with homophilic EpC tetramer cell-cell adhesion.

Cld7 additionally could hamper EpC-mediated cell-cell adhesion by supporting TACE-dependent EpC cleavage [39]. In fact, the TACE inhibitor TAPI strengthened cell-cell adhesion (Figure 3b). TAPI abolished EpEx generation and EpEx recovery was reduced in the supernatant of HEK-EpC^mAG^-cld7 and HEK-EpC-cld7^mPalm^ cells (Figure 3c). Confocal microscopy showed proximity of TACE to EpC only in HEK-EpC-cld7 cells, but very weak colocalization in HEK-EpC^mAG^-cld7 and HEK-EpC-cld7^mPalm^ cells. Cld7 also colocalized with TACE in HEK-EpC^mAG^-cld7, but poorly in HEK-EpC-cld7^mPalm^ cells (Figure 3d). WB confirmed partial recovery of TACE in GEM of HEK-EpC-cld7 and HEK-EpC^mAG^-cld7, but not HEK-EpC-cld7^mPalm^ cells (Figure 3e). Finally, anti-TACE co-immunoprecipitates EpC and cld7 in light sucrose density gradient fractions of HEK-EpC-cld7, but not HEK-EpC cld7^mPalm^ lysates (Figure 3f).

Taken together, cld7 palmitoylation prohibits engagement in TJ and interferes with EpC homophilic cell-cell adhesion. Cld7-associated TACE, which supports EpC cleavage, additionally affected EpCAM cell-cell adhesion.

### Claudin-7 palmitoylation promotes cell motility

**Figure 4:**
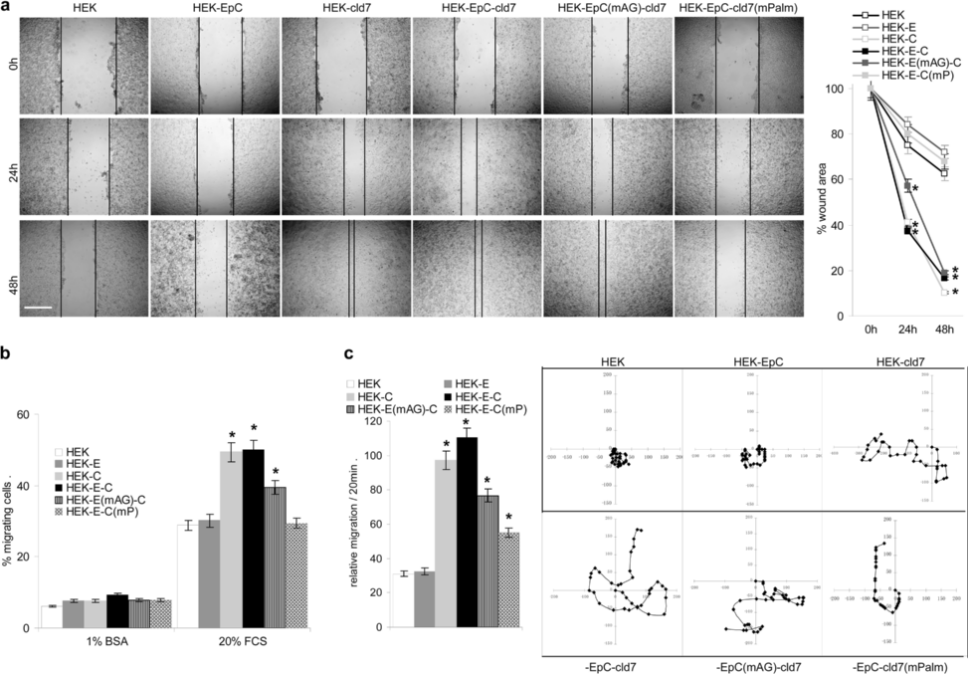
*Palmitoylated claudin7 promotes cell motility* **(a)** Cells were seeded in FN-coated 24-well plates. Subconfluent cultures were scratched with a pipette tip. Wound healing was followed by light microscopy. Examples and mean ± SD (triplicates) of wound closure are shown (scale bar: 250 μm). **(b)** Cells suspended in RPMI / 1%BSA were seeded in the upper chamber of a Boyden chamber. The lower chamber contained RPMI/20%FCS. After 16 h of incubation, cells at the lower membrane site were stained with crystal violet and OD 595 nm was determined after lysis; mean ± SD of triplicates. **(c)** Cells were seeded on BSA-coated 24-well plates. Cell migration was followed for 12 h by videomicroscopy taking pictures every 20 min; examples and the mean ± SD migration of 20 individual cells. (a-c) Significant differences between HEK, HEK-EpC, HEK-EpC-cld7, HEK-EpC-cld7, HEK-EpC^mAG^-cld7 and HEK-EpC-cld7^mPalm^: *. Palmitoylated cld7 significantly strengthens HEK cell migration.

Based on the finding that palmitoylation hampers cell-cell adhesion, we speculated that it might foster motility. The impact of cld7 palmitoylation on motility was evaluated in an *in vitro* wound healing assay, by transwell migration and by videomicroscopy. Wound closure after scratching a cell monolayer is accelerated in HEK-cld7 and HEK-EpC-cld7 compared to HEK and HEK-EpC cells. Motility of HEK-EpC^mAG^-cld7 is comparable to that of HEK-EpC-cld7, but motility of HEK-EpC-cld7^mPalm^ is reduced (Figure 4a). Transwell migration (Figure 4b) and videomicroscopy (Figure 4c) confirmed that HEK-cld7 and HEK-EpC-cld7 cells migrate significantly faster than HEK and HEK-EpC cells and that migration of HEK-EpC-cld7^mPalm^ is reduced. These findings pointed towards palmitoylated cld7 actively promoting motility, but also pointed towards a contribution of EpC. The latter suggestion derives from the observation that HEK-EpC^mAG^-cld7 cells migrate slower than HEK-EpC-cld7 cells.

**Figure 5:**
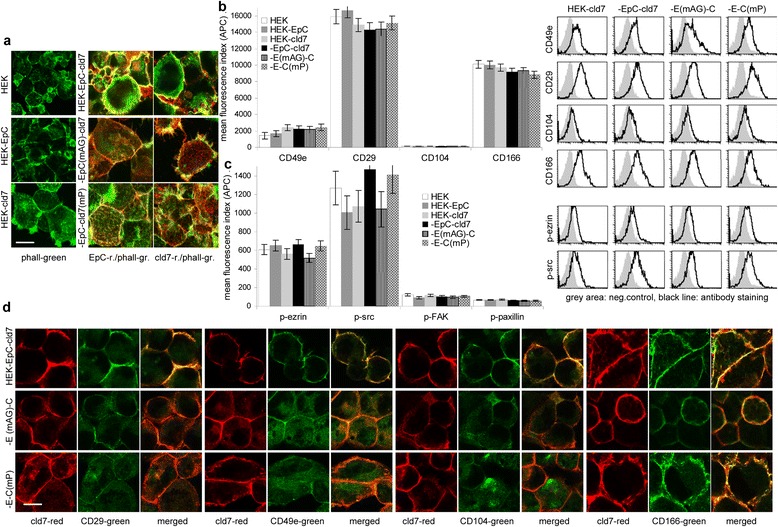
*Cooperativity of palmitoylated claudin-7 with integrins* **(a)** Confocal microscopy of F-actin staining and co-localization (overlay of green and red fluorescence) with EpC and cld7 (scale bar: 10 μm). **(b, c)** Flow cytometry analysis of adhesion molecules and cytoskeletal linker proteins in untransfected and transfected HEK cells; the mean fluorescence index (% stained cells x mean intensity of staining ± SD; three assays) and representative examples are shown. **(d)** Cld7 co-localization with integrins and CD166 (confocal microscopy, green and red fluorescence and overlays, scale bar: 10 μm). Cld7 expression allows for actin filament formation in HEK cells, which likely is supported by the association of cld7 with integrins. Cld7 palmitoylation also facilitates colocalization with CD166.

Phalloidin staining of HEK cells uncovered a disorganized cytoskeleton [57], but a striking enrichment in barbs and strong submembrane bundles in HEK-cld7 and HEK-EpC-cld7 cells. Actin is less efficiently organized in HEK-EpC^mAG^-cld7 and HEK-EpC-cld7^mPalm^ cells (Figure 5a).

**Figure 6:**
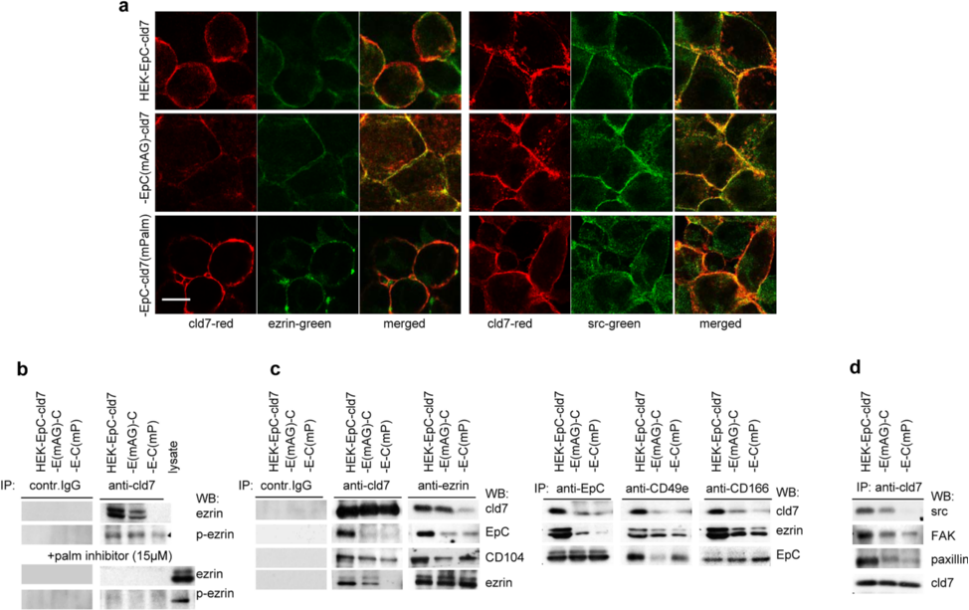
*Cooperativity of palmitoylated claudin-7 with the cytoskeleton* **(a)** Cld7 co-localization with ezrin and src (confocal microscopy, green and red fluorescence and overlays, scale bar: 10 μm) and **(b)** control IgG and cld7 co-immunoprecipitation with ezrin and p-ezrin in the absence or presence of a palmitoylation inhibitor (2-BP, 15 μM) and **(c)** control IgG, cld7 and ezrin co-immunoprecipitation with cld7, EpC, CD104 and ezrin; EpC, CD49d and CD166 co-immunoprecipitation with cld7, ezrin and EpC; **(d)** cld7 co-immunoprecipitation with src, FAK, paxillin and cld7 in transfected HEK cells. Cld7 associates with the cytoskeletal linker protein ezrin. Cld7 palmitoylation facilitates ezrin binding (and F-actin formation). This also accounts for the association with CD49e, CD166, src, FAK and paxillin. Recruitment of the latter two towards cld7 does not essentially depend on, but becomes strengthened by cld7 palmitoylation.

HEK cells express the α5β1 and very weakly the α6β4 integrin; they highly express CD166. Expression remains unchanged in cld7 transfected cells. Furthermore, (p)-ezrin, (p)-src, (p)-FAK and paxillin expression is not significantly reduced in HEK-EpC^mAG^-cld7 and HEK-EpC-cld7^mPalm^ cells (Figure 5b, 5c). Despite unaltered expression, colocalization of cld7 with integrins, but not with CD166, is weakened in HEK-EpC-cld7^mPalm^ and HEK-EpC^mAG^-cld7 (Figure 5d) and colocalization with ezrin and src is weakened in HEK-EpC-cld7^mPalm^ cells (Figure 6a). These findings suggested that the cld7-initiated organization of the actin cytoskeleton (Figure 5a) is supported by ezrin, which associates with palmitoylated cld7. In fact, ezrin poorly associates with cld7^mPalm^ and the association is nearly abolished in the presence of a palmitoylation inhibitor (Figure 6b). As actin reorganization is not completely abolished in HEK-EpC-cld7^mPalm^ cells, we speculated that the cld7-ezrin association is not a direct protein interaction, but is promoted by cld7-associated partner molecules. Besides with EpC, cld7 co-immunoprecipitates with CD104, CD49e and CD166. Co-immunoprecipitation of cld7 with CD49e and CD166 is very weak in HEK-EpC^mAG^-cld7 and HEK-EpC-cld7^mPalm^ lysates. In anti-ezrin precipitates, recovery of EpC and cld7 is most strongly affected in HEK-EpC-cld7^mPalm^ and in anti-CD49e and CD166 precipitates ezrin recovery is strongly reduced in HEK-EpC-cld7^mPalm^ (Figure 6c). These findings indicate that palmitoylated cld7 supports integrin activation. Activated integrins, in turn, associate via phosphorylated ezrin with the actin cytoskeleton. Supporting the mediator role of palmitoylated cld7, src did not co-immunoprecipitate with cld7 in HEK-EpC-cld7^mPalm^ lysates. Co-immunoprecipitation of FAK and paxillin was not abolished, though reduced (Figure 6d). Thus, GEM-located palmitoylated cld7 supports motility via the association with ezrin and integrins, where integrin activation contributes to increased motility.

**Figure 7:**
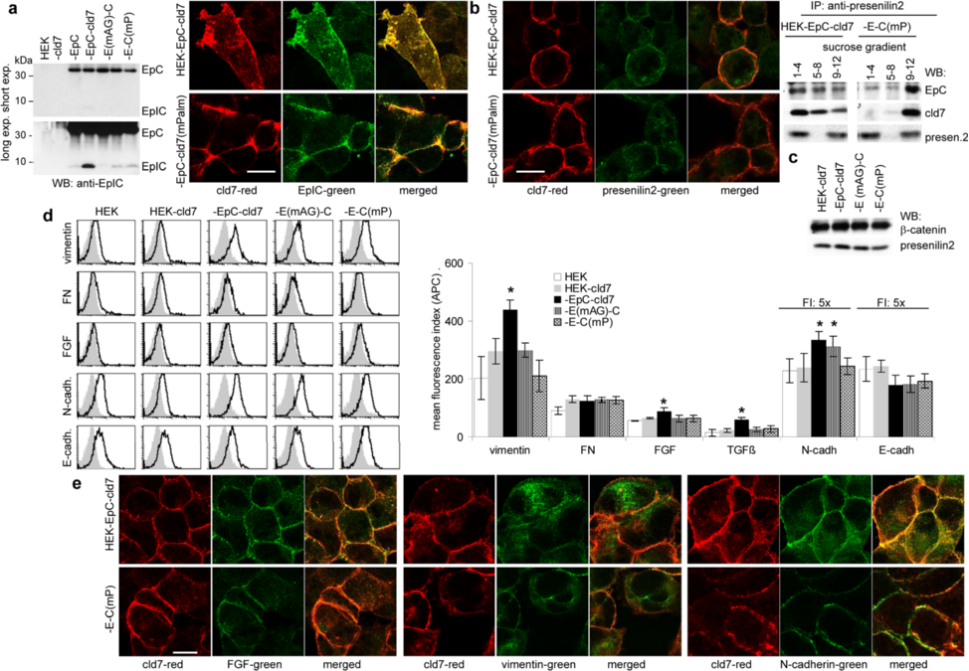
*Palmitoylated claudin-7 supports the generation of EpIC* **(a)** Confocal microscopy of HEK-EpC-cld7 and HEK-EpC-cld7^mPalm^ cells stained with anti-EpIC and -cld7; single fluorescence staining and overlays are shown (scale bar: 10 μm) and WB of lysates of PMA-stimulated transfected HEK cells with anti-EpIC; EpIC and EpC bands are indicated. **(b)** Confocal microscopy of HEK-EpC-cld7 and HEK-EpC-cld7^mPalm^ cells stained with anti-presenilin2 and -cld7 (single fluorescence and overlays, scale bar: 10 μm); sucrose gradient fractions of lysates of PMA-stimulated HEK-EpC-cld7, HEK-EpC^mAG^-cld7 and HEK-EpC-cld7^mPalm^ cells were immunoprecipitated with anti-presenilin2, precipitates were blotted with anti-EpC, anti-cld7 and anti-presenilin2. **(c)** WB of presenilin2 and β-catenin in PMA-stimulated transfected HEK cells. **(d)** Flow cytometry analysis of mesenchymal proteins in PMA-stimulated transfected HEK cells; the mean fluorescence index (% stained cells x mean intensity of staining ± SD; three assays) and representative examples are shown (mean % stained ± SD, mean intensity ± SD; triplicates and representative examples); significant differences between HEK-cld7 versus HEK-EpC-cld7, HEK-EpC^mAG^-cld7 and HEK-EpC-cld7^mPalm^ cells: *. **(e)** Representative examples of FGF, vimentin and N-cadherin co-localization with cld7 (confocal microscopy, scale bar: 10 μm). Palmitoylated cld7 facilitates the generation of EpIC by associating with presenilin-2 in GEM. EpIC generation is accompanied by a slight upregulation of mesenchymal protein expression.

Videomicroscopy pointed towards an additional contribution of EpC to cld7-promoted motility (Figure 4c). Cld7 contributes via the association with TACE to EpC cleavage (Figure 3f). As presenilin2 also resides in GEM [58], we speculated that the association of EpC with cld7 might facilitate the release of EpIC. Staining with anti-cld7 and anti-EpIC revealed, expectedly, stronger colocalization in the membrane of HEK-EpC-cld7 than HEK-EpC-cld7^mPalm^ cells, but also more pronounced recovery of free EpIC in the cytoplasm. WB confirmed higher EpIC recovery in HEK-EpC-cld7 than in HEK-EpC lysates. On the other hand, EpIC was not detected in HEK-EpC^mAG^-cld7 and very weakly in HEK-EpC-cld7^mPalm^ lysates (Figure 7a). This implies that palmitoylated cld7-associated, GEM-located EpC becomes particularly susceptible for presenilin2. Nonetheless, presenilin2 co-immunoprecipitated also with cld7^mPalm^, but exclusively in heavy fractions (Figure 7b). Flow cytometry and WB confirmed unaltered β-catenin expression in HEK-cld7, −EpC-cld7, −EpC^mAG^-cld7 and -EpC-cld7^mPalm^ cells and only a minor reduction of presenilin2 in HEK-EpC-cld7^mPalm^ cells (Figure 7c, Additional file 4). Increased EpIC recovery in HEK-EpC-cld7 cells was accompanied by slightly upregulated expression of vimentin, fibroblast growth factor (FGF), TGFβ and N-cadherin. Upregulated N-cadherin expression did not correlate with the generation of EpIC (HEK-EpC-cld7) and downregulation of E-cadherin in HEK-EpC-cld7 cells did not reach the level of statistical significance. Colocalization of FGF, N-cadherin and vimentin is less pronounced in HEK-EpC-cld7^mPalm^ than HEK-EpC-cld7 cells (Figure 7d, 7e), where palmitoylated cld7 supports EpIC generation. The pronounced EpIC generation by palmitoylated cld7 might strengthen expression of mesenchymal markers associated with a migratory phenotype. However, our findings point towards an engagement of additional components.

Taken together, cld7 promotes motility, which is partly due to the association of palmitoylated cld7 with p-ezrin and is supported by the association with integrins and CD166. Palmitoylated cld7 promotes the generation of EpIC, which contributes to motility by forcing mesenchymal protein expression.

### Palmitoylated claudin-7 supports invasiveness

Cld7 repressing MMP3 [43], we wondered whether HEK-cld7 cells gain in motility, but concomitantly loose invasiveness.

**Figure 8:**
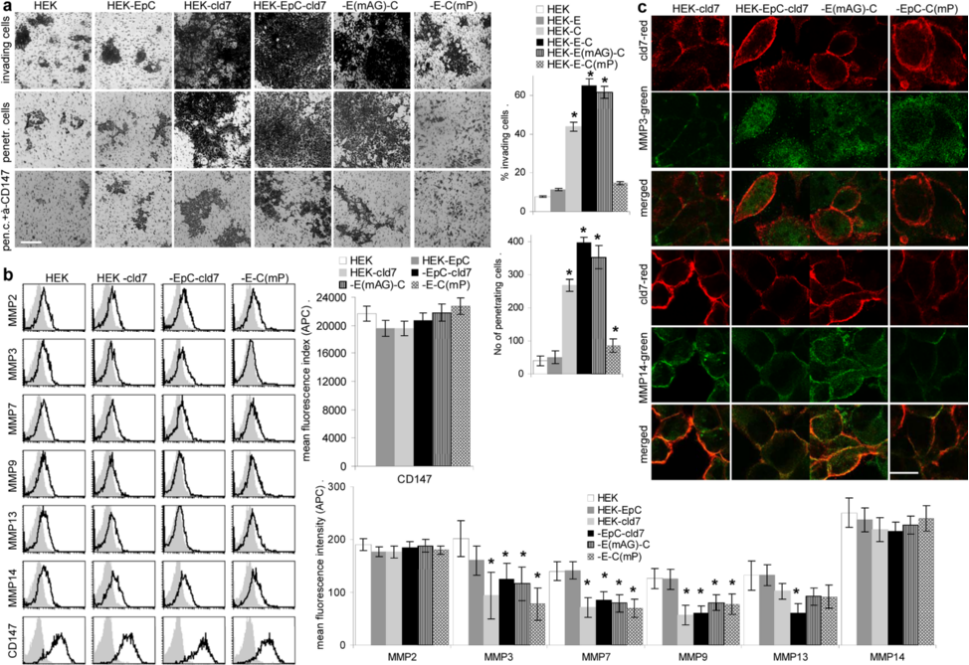
*Palmitoylated claudin-7 and invasion* **(a)** HEK cells were seeded in matrigel-coated transwell plates, matrigel invasion (recovery within the matrigel) and penetration (recovery at the lower site of the insert) was evaluated after 16 h; mean number ± SD (triplicates) of invading and penetrating cells and representative examples (scale bar: 250 μm); significant differences between HEK-EpC-cld7 and HEK-EpC^mAG^-cld7 or HEK-EpC-cld7^mPalm^: *. **(b)** Flow cytometry of MMP and CD147 expression in untransfected and transfected HEK cells; the mean fluorescence index (% stained cells x mean intensity of staining ± SD; three assays) and representative examples are shown; significant differences in transfected versus non-transfected HEK cells: *. **(c)** Confocal microscopy of transfected HEK cells stained with anti-cld7 and anti-MMP3 or -MMP14; single fluorescence and overlays of representative examples (scale bar: 10 μm). Despite slight downregulation of MMP3, MMP7 and MMP9 by cld7, palmitoylated cld7 promotes invasiveness.

HEK-cld7, HEK-EpC-cld7 and HEK-EpC^mAG^-cld7 cells show pronounced matrigel invasion. Invasion of HEK-EpC-cld7^mPalm^ resembles that of HEK, i.e. is strongly reduced (Figure 8a). This expectation-opposing finding prompted a search for MMP activity of untransfected and transfected HEK cells. HEK cells express MMP2, MMP3, MMP7, MMP9 and MMP13 at low, MMP14 at intermediate and CD147 at high level. MMP3, MMP7 and MMP9 expression is slightly reduced in cld7 expressing cells, but the reduction does not essentially correlate with cld7 palmitoylation (Figure 8b). Notably, cld7 does not colocalize with MMP3 and only palmitoylation-competent cld7 weakly colocalizes with MMP14 (Figure 8c).

**Figure 9:**
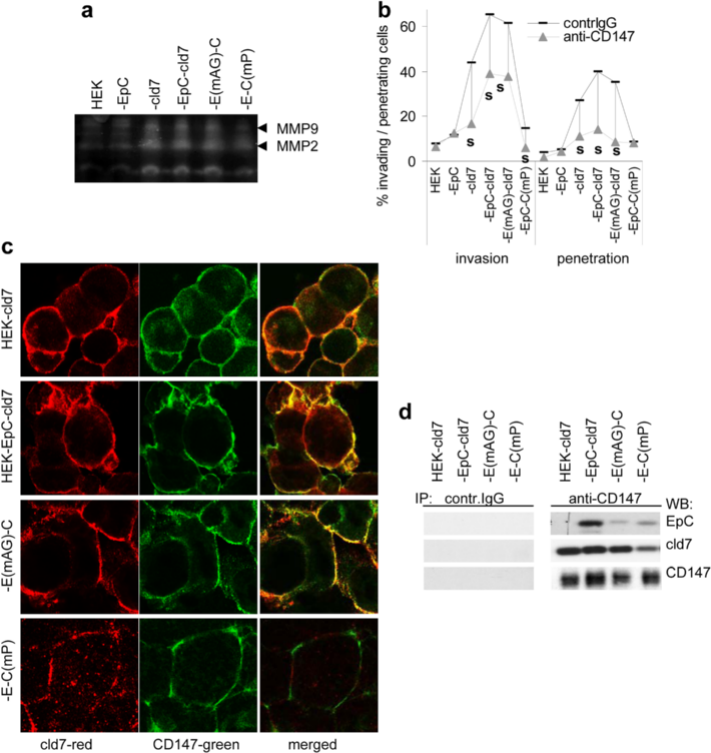
*Palmitoylated claudin-7, MMP activity and CD147* **(a)** Zymography of HEK, HEK-EpC, HEK-cld7, HEK-EpC-cld7 and HEK-EpC^mAG^-cld7 or HEK-EpC-cld7^mPalm^ supernatants. **(b)** HEK cells were seeded in matrigel-coated transwell plates in the presence of anti-CD147 (10 μg / ml) (a representative example is included in Figure 8a); invasion and penetration was evaluated as above; significant inhibition by anti-CD147: **s. (c)** Confocal microscopy of HEK-cld7, HEK-EpC-cld7 and HEK-EpC^mAG^-cld7 or HEK-EpC-cld7^mPalm^ cells stained with anti-cld7 and anti-CD147 (single fluorescence and overlays, scale bar: 10 μm). **(d)** HEK-cld7, HEK-EpC-cld7, HEK-EpC^mAG^-cld7 and HEK-EpC-cld7^mPalm^ lysates were precipitated with control IgG or anti-CD147; after SDS-PAGE and transfer precipitates were blotted with anti-EpC, anti-cld7 and anti-CD147. Palmitoylated cld7-promoted invasiveness likely is supported by associated MMP14 and CD147, which can strengthen MMP2 and MMP9 activation and expression.

As changes in protease expression could not explain increased invasiveness of HEK-EpC-cld7 cells, we speculated on pronounced protease activation, possibly due to the weak association of cld7 with MMP14. Zymography revealed low MMP2 and MMP9 activity in HEK and HEK-EpC cells, strongly increased activity in HEK-EpC-cld7, but reduced activity in HEK-EpC-cld7^mPalm^ cells **(**Figure 9a). Though this finding fits reduced invasiveness of HEK-EpC-cld7^mPalm^, we wondered, whether palmitoylated cld7 recruits CD147 (EMMPRIN) into GEM, which could contribute to MMP stabilization. Indeed, anti-CD147 efficiently inhibits matrigel invasion of HEK-cld7, HEK-EpC-cld7 and HEK-EpC^mAG^-cld7 cells (Figure 9b). Furthermore, CD147 colocalizes and co-immunoprecipitates with cld7 in HEK-EpC-cld7 and HEK-EpC^mAG^-cld7, but hardly in HEK-EpC-cld7^mPalm^ cells (Figure 9c, 9d, Additional file 3).

Though slightly affecting MMP3, MMP7 and MMP9 expression, cld7 fosters invasiveness via GEM-located MMP14, which holds MMP2 and MMP9 in membrane proximity. High CD147 expression likely promotes MMP induction.

In brief, palmitoylated cld7 poorly associates with TJ proteins. Instead, it is recruited into GEM, where it associates with the cytoskeleton, integrins, and integrin signaling partners, EpC and proteases. It promotes integrin and protease activation and EpC cleavage, supporting motility and invasion.

## Discussion

Functional activities of claudins outside TJ are not well explored. This includes cld7, highly expressed in the upper gastrointestinal tract and the liver, where it is not exclusively found in TJ [6,8,9,28,29,59]. Instead, cld7 associates in hepatocyte progenitors and gastrointestinal tumors with EpC that is not a TJ component [36,40,59,60]. Taking the importance of cld7 in organogenesis, cld7^ko^ mice dying few days after birth [43], it becomes demanding to unravel TJ-independent cld7 activities. We approached the question of cld7 localization using easily transfectable HEK cells to generate a tool of cells stably expressing wt or mutated cld7 and/or EpC. Whereas single serine to alanine mutations was ineffective, a palmitoylation site mutation was accompanied by a striking shift in the membrane subdomain localization. Thus, we focused on palmitoylation defective cld7 and on mutated EpC that cannot bind cld7 [61]. The latter allowed differentiating between genuine cld7 activities and those requiring the association with EpC.

### Cld7 palmitoylation and tight junctions

In advance of discussing the impact of cld7 palmitoylation on membrane subdomain localization, it should be remembered that mutating AA184 and AA186 efficiently prevented cld7 palmitoylation and that palmitoylation at the C-terminal tail appears to be dominating, because only a very faint palmitoylation signal was detected in HEK-EpC-cld7^mPalm^. The high efficacy of cld7^mPalm^ relocalization and striking differences in associating molecules are in line with the dominance of the C-terminal tail cld7 palmitoylation.

Palmitoylated cld7 is enriched in GEM, but is not recovered in TJ protein complexes. Cld7 poorly colocalizes and co-immunoprecipitates with cld3, cld4 and ZO-1, which strikingly differs from colocalization and co-immunoprecipitation of ZO-1 with cld3 and cld4. Instead, palmitoylation-deficient cld7 associates with TJ proteins. We have not explored, whether overexpression of cld7^mPalm^ in HEK cells suffices establishing classical TJ, as was described for a cld7 overexpressing epithelial like cell line [62]. However, HEK-EpC-cld7^mPalm^ cells do not form tightly packed monolayers like epithelial cells and trogocytosis of membrane fractions from biotinylated HEK-EpC-cld7^mPalm^ by non-biotinylated HEK-EpC-cld7^mPalm^ was not in support of “kissing points” (data not shown). On the other hand, only palmitoylated cld7 associates with EpC.

Palmitoylation supports anchoring in GEM [16,17]. GEM microdomains are signaling platforms facilitating attachment of cytoplasmic kinases [16,18,19]. GEM also are prone for internalization [21-24]. This is a sequel of the attachment of complexes that promote scission, fission and early endosome traffic towards multivesicular bodies, where proteins become ubiquitinated and degraded in the proteasome [63]. Alternatively, the multivesicular bodies are transported back to the plasma membrane and the intraluminal vesicles are released as exosomes [64]. Notably, claudins were recovered in internal vesicles colocalizing with syntaxin4 [65], but did not reintegrate into TJ. Syntaxin4 being one of the components for vesicular transport [66], we suggest an independent route of trafficking of TJ- versus GEM-integrated claudins. In fact, colon carcinoma lines secrete two populations of exosomes, only exosomes from the basolateral region contain the EpC-cld7 complex [59]. Furthermore, the association with EpC hampers the integration of cld7 and via cld7 the integration of cld1 into TJ [60].

Palmitoylation-deficiency of cld7 had significant bearing on associating molecules and their activation state. A strikingly reduced number of proteins co-immunoprecipitated with cld7 in lysates of HEK-EpC-cld7^mPalm^ and phosphorylation of co-immunoprecipitating proteins was additionally impaired. The number of molecules co-immunoprecipitating with anti-cld7 from HEK-EpC^mAG^-cld7 lysates was also reduced, though the reduction was less pronounced. These findings indirectly confirm the direct association of EpC with cld7 [35] and support a dominating role of palmitoylation-competent cld7 in associated molecule activation. It is particularly worthwhile noting that only palmitoylation-competent cld7 abundantly associates with vesicular transport molecules. It remains to be explored, whether this translates into a central role of cld7 in exosome composition and release.

Taken together, the membrane subdomain localization of cld7 is dictated by palmitoylation. This has most severe consequences on associating molecules and their activation state. Building on this, we asked for functional consequences.

### Palmitoylated claudin-7 prohibits adhesion and supports motility

The striking impact of cld7 palmitoylation on the redistribution into GEM and on the association with (phosphorylated) GEM-located molecules suggests that palmitoylated cld7 fulfills TJ-independent activities. We had described that tetramer formation and homophilic EpC binding [67] is distorted by the association with cld7 [56]. We now reported that the cld7-mediated inhibition of homophilic EpC binding depends on cld7 palmitoylation and the GEM localization. The elegant study by Wu et al. [60] also reported that EpC interferes with the integration of cld1 and cld7 in TJ and supports recruitment into vesicles. These findings are in line with our observations, which suggest that cld7 palmitoylation is the prime cause, followed by the association with EpC. GEM-located TACE [68], which cuts transmembrane molecules in membrane proximal regions [69], supports the interference of cld7 with homophilic EpC binding by the liberation of EpEx. Thus, palmitoylated cld7 inhibits EpC-mediated cell-cell adhesion by prohibiting tetramer formation and by promoting EpEx liberation.

Palmitoylated cld7 also actively supports cell motility that is accompanied by organization of the disordered actin cytoskeleton in HEK cells [57]. GEM-located, palmitoylated cld7 associates with CD166 and α5, the latter being the most abundantly expressed integrin in HEK cells [57]. The cld7 colocalization with α5 and CD166 in GEM suffices for src, FAK and paxillin association and phosphorylation. Importantly, palmitoylated cld7 associates with phosphorylated ezrin, which can explain the striking consequences on HEK cell motility and actin cytoskeleton organization. As outlined above, cld7 also cooperates with TACE, which also cleaves the cell-matrix adhesion molecule CD166 [39,70,71]. It remains to be explored, whether cleavage of CD166 indirectly supports motility by breaking adhesive cell contacts.

Palmitoylated cld7 additionally contributes to motility by facilitating EpIC generation. By a change in conformation after EpEx cleavage, presenilin2 can bind and liberate the cotranscription factor EpIC [39]. Though EpIC generation may not essentially depend on cld7 coexpression [39], EpC recruitment into GEM via palmitoylated cld7 strengthens the efficacy of EpIC generation, which is accompanied by increased expression of the mesenchymal proteins FGF, TGFβ and N-cadherin. The shift towards mesenchymal protein expression could strengthen motility.

Though the association of palmitoylated cld7 with EpC does not suffice for a full-fledged shift towards EMT, our findings are quite distinct from a recent publication on cld7-induced MET, which is accompanied by rab25 upregulation [72]. Rab25 can be a tumor suppressor in colon cancer [73], but promotes tumor growth in several different cancer types [74]. Interestingly, in hormone receptor negative breast cancer rab25 expression is very low, but the small subpopulation of cancer initiating cells express rab25 [75]. Based on this finding, we suggest that palmitoylated cld7 and the vesicle transporter rab25 support cancer-initiating cell activities, possibly via the transfer of exosomes. Our suggestion is in line with the central importance of rab molecules in vesicle traffic and exosome release [76], the delivery of cld7-EpC complex expressing exosomes by colorectal cancer cells and the abundant association of vesicle traffic-associated molecules with palmitoylation-competent cld7.

Despite the need to experimentally support the latter hypothesis, our data demonstrate an active contribution of palmitoylated cld7 to cell motility, which is based on the GEM-location-dependent association with EpC, integrins, CD166, cytoskeletal linker and signaling molecules.

### Claudin7 and proteases

Cld7 can repress MMP3 [43], which could hamper invasiveness. Opposing our expectation, HEK-cld7 cells are strongly invasive. HEK cells express MMP3 and MMP7 at an intermediate level. Independent of cld7 palmitoylation, both proteases are slightly downregulated in HEK-cld7 cells. Cld7 expression did not affect expression of other MMPs in HEK cells. On the other hand, gelatinolytic activity of HEK-cld7 cells was increased. Palmitoylated cld7 colocalizes with MMP14, which supports retention of MMP2 and MMP9 close to the plasma membrane. Furthermore, HEK cells highly express CD147 that stimulates MMP expression in neighboring cells [77,78]. CD147 colocalizes and co-immunoprecipitates with palmitoylated cld7 and thereby can contribute to cld7-promoted invasiveness. This interpretation is supported by the finding that anti-CD147 strongly inhibits HEK-cld7 and HEK-EpC-cld7, but not weak HEK-EpC-cld7^mPalm^ cell invasion.

Taken together, in HEK cells a repression of MMP3 by cld7 has no bearing on invasiveness, as palmitoylated cld7 supports invasion by associating with MMP14 and CD147.

## Conclusions

The (patho)physiological relevance of the EpC-cld7 complex was demonstrated repeatedly, most convincing by the death of EpC^ko^ mice [41,42], by the shift of cld1 and cld7 towards TJ in EpC^kd^ cells [60], by the pronounced recovery of GEM-located cld7-EpC-CD44v6-Tspan8 complexes in colorectal cancer [36,52] and by the metastasis-supporting activity of the EpC-cld7 complex in pancreatic and colorectal cancer [35,36,38,52,61]. We here demonstrated that under physiological conditions, i.e. without chemical or antibody crosslinking, a considerable part of cld7 is palmitoylated and that palmitoylation drives cld7 into GEM, but prohibits integration into TJ. In GEM, palmitoylated cld7 associates with adhesion molecules, proteases, cytoskeletal linker and signal transduction molecules, which supports motility and invasion. Though the reason for the preferential palmitoylation state of cld7 remains to be explored, palmitoylated cld7 exhibits TJ opposing activities, which should be taken into account considering the role of cld7 in development and metastasis.

## Material and methods

### Cell lines and transfectants

HEK293 cells [79] cells were transfected with rat EpC cDNA and cotransfected with rat cld7 cDNA using the pcDNA3.1 vector that carries either the neomycin or the hygromycin resistance cDNA. Where indicated, the cld7 cDNA was mutated at the serine residues AA33, AA69, AA87, AA172, AA204, AA206 and AA207, exchanging serine by alanine (HEK-EpC-cld7^mSxx^) and with cld7 cDNA, where the palmitoylation site was destroyed by mutations at AA184 and AA186 (HEK-EpC-cld7^mPalm^). The EpC cDNA was mutated at AA279 and AA282, which are located in the transmembrane region and account for the EpC-cld7 association (HEK-EpC^mAG^-cld7) (Additional file 1) and cDNA inserts were sequenced. Cells were transfected with lipofectamin and cultured in RPMI/10% FCS plus 500 μg/ml G418 and/or 150 μg/ml hygromycin. Transfection efficacy, evaluated by WB and flow cytometry, ranged between >90% to 98%.

### Antibodies and chemicals

Additional file 5.

### Sucrose density gradient centrifugation

Cell lysates in 2.5 M sucrose were overlaid by a continuous sucrose gradient (0.25 M-2 M) and centrifuged (15 h, 150000 g) and twelve 1 ml fractions were collected. Proteins located in GEM are recovered in fractions 2–4.

### *In vitro* kinase assay

Immune complexes were suspended in lysis buffer containing a protease inhibitor mix. After centrifugation, beads were resuspended in 30 μl kinase assay buffer, 10 μCi [^32^P]γ-ATP and incubated (15 min, 37°C), stopping the reaction by 10 μl non-reducing 6x Laemmli buffer. SDS-PAGE was followed by autoradiography.

### IP, Western blot (WB)

Lysates (30 min, 4°C, HEPES buffer, 1% Lubrol or 1% TritonX-100, 1 mM PMSF, 1 mM NaVO_4_, 10 mM NaF, protease inhibitor mix) were centrifuged (13000 g, 10 min, 4°C), mixed with antibody (1 h, 4°C) and incubated with ProteinG-Sepharose (1 h). For the analysis of released EpEx (EpC extracellular domain), culture supernatant were depleted of exosomes [80]; supernatant were 10-times concentrated. Washed complexes/lysates, dissolved in Laemmli buffer, were resolved in 10%-12% SDS-PAGE. For the recovery of EpIC, Tris-Tricine and 16% SDS-PAGE was used under non-reducing condition. After protein transfer, blocking, blotting with antibodies, blots were developed with ECL.

### Palmitoylation assay

Palmitoylation of cld7 was determined using the IP-ABE method [81]. In brief, cultured cells were lysed in the presence of N-ethylmaliemide (NEM), using lysis buffer (LB) (50 mM NEM, 1% Triton-X-100, 1x protease inhibitor cocktail, 1 mM phenylmethanesulfonylfluoride (PMSF)) for irreversibly blocking unmodified thiol groups. After an IP with anti-cld7, G-Sepharose coupled precipitates where incubated with hydroxylamine (HAM) buffer (1 h, room temperature, LB pH7.2, 1 M HAM) for specific cleavage and unmasking of the palmitoylated cysteine thiol group. Thereafter samples were incubated in biotin-BMCC buffer (LB pH 6.2, 1 μM biotin-BMCC, 1 h, 4°C) for selective labeling of the palmitoylated cysteines. Samples were eluted in LB pH7.5 followed by SDS-PAGE and WB with streptavidin-HRP.

### MALDI-TOF analysis

After SDS-PAGE, gels were stained. Protein digestion, sample preparation, MALDI-TOF fingerprint analysis, post-source decay fragmentation analysis and database searches were performed as described [82].

### Flow-cytometry

Flow-cytometry followed routine procedures. In brief, 1–2.5×10^5^ cells were seeded in 96-well plates. After washing with PBS/1% BSA, cells were incubated with the primary antibody (2–10 μg/ml, 40 μl, 30 min, 4°C). Cells were washed 3-times with PBS/1% BSA and incubated with a secondary dye-labeled antibody at predetermined concentration. Negative controls were only incubated with the secondary dye-labeled antibody (40 μl, 30 min, 4°C). For intracellular staining, cells were fixed (PBS/1% formaldehyde, 30 min, 4°C) and permeabilized (PBS/0.5% Tween-20, 20 min, 4°C). Samples were processed in a FACS-Calibur and evaluated with the CellQuest program. Data are presented as a weighted score, the fluorescence index (FI) representing the percentage stained cells × the mean fluorescence intensity above background.

### ELISA

For the recovery of EpEx, cells were cultured in serum-free medium for 48 h. Supernatants were centrifuged, concentrated and adjusted to 100 μg/ml, evaluating the amount of EpEx by a direct ELISA coating the wells with culture supernatant and proceeding with a standard protocol using anti-rat EpC (D5.7) for detection.

### Confocal microscopy

Cells on glass-slides were fixed (4% paraformaldehyde, 20 min on ice), permeabilized (1% Triton-X100, 4 min, on ice), blocked (PBS/1% gelatin, 30 min, on ice), incubated with primary antibody (60 min, on ice), washed, incubated with fluorochrome-conjugated secondary antibody (60 min, on ice), blocked (IgG with irrelevant specificity of the same species as the primary antibody), incubated with a second, dye-labeled primary antibody and washed. Slides were mounted in Elvanol. Digitized images were generated using a Leica LMS780 microscope and the Carl Zeiss Vision software for evaluation. The Z-stack offers 30 positions through the depth of the cell. All pictures were taken at Z-stack 14–16. Depending on the quality of the antibody and the density of marker expression, the intensity for the green channel varied between 700–900 master gain values and for the red channel between 500–750 master gain values. The photosystem automatically generates the single fluorescence and overlay pictures.

### Cell adhesion

CFSE-labeled cells were seeded on a cell monolayer in 96-well plates. After washing, adherent cells were lysed, evaluating fluorescence intensity photometrically. Adhesion is presented as percent seeded cells.

### Migration

Cells, in the upper part of a Boyden chamber (RPMI / 0.1% BSA), were separated from the lower part (RPMI / 20% FCS) by 8 μm pore size polycarbonate-membranes. After 16 h, the lower membrane side was stained (crystal-violet), measuring OD595 after lysis. Migration is presented as percentage input cells. In an *in vitro* wound healing assay, a subconfluent monolayer was scratched with a pipette tip. Wound closure was controlled by light microscopy. For videomicroscopy, cells (5×10^4^) were seeded on matrix protein-coated 24-well plates. Plates were placed under an Olympus IX81 inverse microscope with an Hg/Xe lamp, an incubation chamber (37°C, 5% CO_2_), a CCD camera (Hamamatsu) and a ScanR acquisition soft ware (Olympus, Hamburg, Germany). Two pictures (20-fold magnification)/chamber (2 ms exposure) were taken every 20 min for 12 h. Migration was quantified according to Manual_tracking plugin running in the open-source software Image J.

### Statistics

All experiments were repeated at least 3 times. Mean values of triplicates or for videomicroscopy of 20 individual cells or of, at least 3 independent experiments are presented. Significance was evaluated by the two tailed Student’s *t*-test. P-values <0.05 were considered significant.

## Additional files

Additional file 1:
**Primers.**
Additional file 2:
**The impact of cld7 serines on membrane subdomain localization and the association with EpCAM.**
Additional file 3:
**MALDI-TOF analysis of molecules co-immunoprecipitating with cld7.**
Additional file 4:
**The impact of cld7 palmitoylation on cld7 phosphorylation, presenilin2 and β-catenin expression.**
Additional file 5:
**Antibodies and chemicals.**

## Abbreviations

cld7: claudin-7
cld7^mPalm^: cld7 with a mutated palmitoylation site
cld7^mSxx^: cld7 with serine exchange by alanine at the indicated AA
EpC: EpCAM
EpC^AG^: EpC with a point mutation at AA279 and AA282, which prohibits cld7 binding
EpEx: EpC extracellular domain
EpIC: EpC intracellular domain
GEM: glycolipid-enriched membrane domains
HEK: HEK293 cells
HEK-EpC: HEK cells transfected with EpC
HEK-cld7: HEK cells transfected with cld7
HEK-EpC^mAG^: HEK cells transfected with EpC^mAG^
HEK-cld7^mPalm^: HEK cells transfected with cld7^mPalm^
m: mutated
palm: palmitoylation
TJ: tight junctions
