# Supplementary material for: The importance of claudin-7 palmitoylation on membrane subdomain localization and metastasis-promoting activities
Source: Cell Commun Signal. 2015 Jun 9;13:29. doi: 10.1186/s12964-015-0105-y (PMC4459675; doi:10.1186/s12964-015-0105-y)
Supplement: Additional file 1: — Primers. [file 12964_2015_105_MOESM1_ESM.pdf]

Additional File 1

Primers

Cld7mS33: for: CTATCCCACAGTGGCAGATGGGCTCCTATGCAGGCGACAAC
rev: GTTGTCGCCTGCATAGGAGCCCATCTGCCACTGTGGGATAG

Cld7mS69: for: GAGCTGCAAAATGTACGACGCGGTGCTTGCCCTGCCAGC
rev: GCTGGCAGGGCAAGCACCGCGTCGTACATTTTGCAGCTC

Cld7mS87: for: CGAGCCTTAATGATTGTGGCCTTGGTGTTGGGCTTCTTG
rev: CAAGAAGCCCAACACCAAGGCCACAATCATTAAGGCTCG

Cld7mS172: for: CTTTATCGGCTGGGCAGGGGCTGCTCTGGTCCTTCTGGG
rev: CCCAGAAGGACCAGAGCAGCCCCTGCCCAGCCGATAAAG

Cld7ΔPalm184: for: GGAGGGGCCCTGCTCTCTTTCCTCCTCCCCCGGCAGTGAAAGCAAAG
rev: CTTTGCTTTCACTGCCGGGGGAGGAGGAAAGAGAGCAGGGCCCCTCC

The primers for cld7mS204, cld7mS206, cld7mS207 and for EpCmutG282 and mutA279 (EpC^{mAG}) are published [1].
