# Supplementary material for: The importance of claudin-7 palmitoylation on membrane subdomain localization and metastasis-promoting activities
Source: Cell Commun Signal. 2015 Jun 9;13:29. doi: 10.1186/s12964-015-0105-y (PMC4459675; doi:10.1186/s12964-015-0105-y)
Supplement: Additional file 2: — The impact of cld7 serines on membrane subdomain localization and the association with EpCAM. [file 12964_2015_105_MOESM2_ESM.pdf]

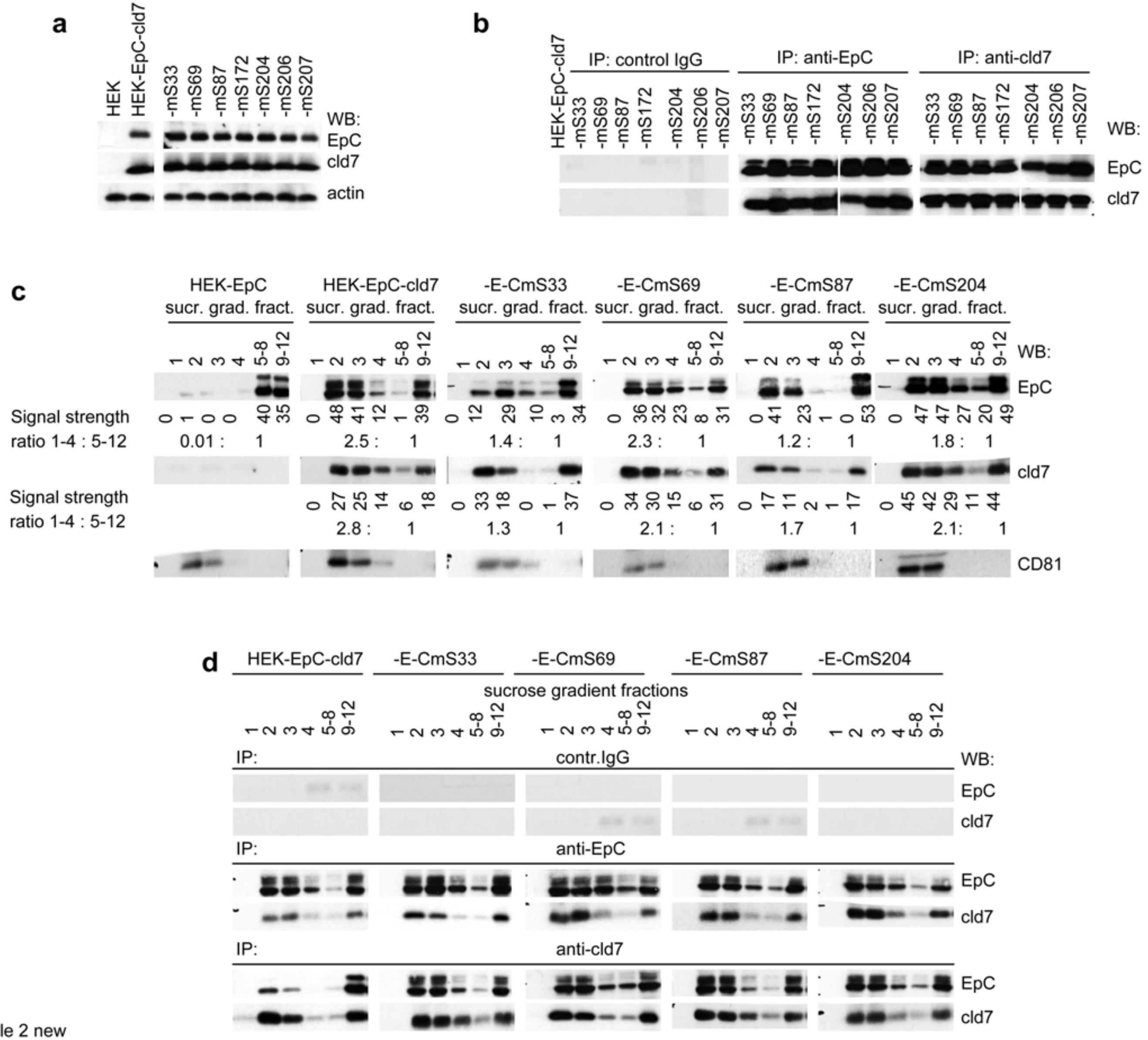

Add. File 2 new

Additional File 2 The impact of cld7 serines on membrane subdomain localization and the association with EpCAM (a-d) HEK cells were transfected with EpC and/or wt cld7 or cld7, where 7 serine phosphorylation sites were exchanged by alanine. (a) WB of HEK and HEK-EpC-cld7^{mS} at the indicated positions with anti-EpC, anti-cld7 and anti-actin as loading control; (b) lysates of cells as above were immunoprecipitated with control IgG, anti-EpC or anti-cld7 and blotted after SDS-PAGE and transfer with anti-EpC and anti-cld7; (c) WB of lysates of some of the cell lines above after sucrose gradient centrifugation. The relative protein recovery in light and heavy density fractions and the ratio of the light density to heavy density proteins are indicated; (d) IP of sucrose gradient fractions of the same cell lines as in (c) with control IgG, anti-EpC and anti-cld7 and WB with anti-EpC and anti-cld7.

Cld7 and co-immunoprecipitating EpC are partly recovered in light density fractions. Serine phosphorylation has no significant impact on cld7 localization in membrane subdomains and EpC co-immunoprecipitation is not significantly affected by exchange of the indicated cld7 serine phosphorylation sites.
