# Supplementary material for: The importance of claudin-7 palmitoylation on membrane subdomain localization and metastasis-promoting activities
Source: Cell Commun Signal. 2015 Jun 9;13:29. doi: 10.1186/s12964-015-0105-y (PMC4459675; doi:10.1186/s12964-015-0105-y)
Supplement: Additional file 3: — MALDI-TOF analysis of molecules co-immunoprecipitating with cld7. [file 12964_2015_105_MOESM3_ESM.pdf]

Additional File 3

MALDI-TOF analysis of molecules co-immunoprecipitating with cld7

		HEK-E-C		-E(mAG)-C		-E-C(mP)		
Protein Description	Da	hits	%Cov	hits	%Cov	hits	%Cov	family / function
14-3-3 proteins	29326	11	49	8	34.5	5	18.4	signal transduction by binding to phosphoserine
28 kDa phosphoprotein	20618	5	22.7	4	30.4	4	22.1	unknown
3-hydroxyacyl-CoA dehydrogenase-2	47602	3	14.6	15	54.3	12	44.4	mitochondrial matrix, oxidation of 3-hydroxyacyl-CoA
Actin	42052	14	55.5	16	61.1	18	66.1	cytoskeletal protein
Act. RNA polymerase II transcr. coactivator	14386	6	56.7	9	57.5	8	62.2	nuclear translocation upon activation
Acyl-coenzyme A thioesterase	15065	1	8.6			4	14.8	oxidation of fatty acids
Alpha-enolase	47481	13	49.5	2	11.5	4	13.4	glycolytic enzyme
Annexins	35971	8	30	6	31	4	19.5	calcium-dependent phospholipid-binding protein
AP-2 complex	17178	1	4.9	3	19	2	6.7	protein coat of coated-vesicles
<u>ATPase 16 kDa & F</u>	15725	1	11.6	1	11.6	1	11.6	organelle acidification for sorting, zymogen activation, endocytosis
BAG2	23928	1	5.2	2	8.5	1	5.2	compete with Hip for Hsc70/Hsp70 ATPase domain
<u>BET1</u>	13395	1	15.3	3	44.1	1	15.3	vesicular transport from ER to Golgi
BolA-like protein 2	10281	1	18.6	1	18.6	1	18.6	unknown
BRI3-binding protein	27932	1	6.8	1	6.8	2	14.3	oncogene binding protein
C14orf166	28165	6	31.6	9	47.1	3	23	unknown
C7orf50	22127	2	23.2	4	35.6	5	41.8	unknown
CAAX prenyl protease 1	55063	1	3.4	2	5.5	1	3.4	metalloproteinase
Calmodulin	16827	4	45.6	3	30.9	3	36.9	phosphorylase kinase
Casein kinase II	45229	6	32	6	27.6	4	11	protein phosphorylation
CASP	77636	1	2.1	1	2.1	1	2.1	caspase, cysteine peptidase
Cell growth-regulating nucleolar protein	44044	1	3.7	1	3.7	3	10.8	metal ion binding
Chromatin target of PRMT1	26380	2	14.5	1	5.2	2	10.5	methyltransferase 1
Cilia- and flagella-associated protein 20	22931	1	10.4	1	6.2	1	6.2	arginine methylation activity
Cleavage and polyadenylation specificity factor5	26268	5	27.3	2	10.6	2	7.5	endonuclease
Coatomer subunit epsilon	34688	7	36.4	1	8.4	6	32.8	non-clathrin-coated vesicles, retrograde Golgi-to-ER transport
Cofilin-1	18719	3	25.3	1	6.6	1	6.6	polymerize / depolymerize F- and G-actin
Coiled-coil domain-containing proteins	25820	4	36.3	8	38.1	5	36.3	actin-binding, cytoskeleton remodeling, migration
CIRBP	18637	5	38.4	5	38.4	4	32	Cold-inducible RNA-binding
Complement C1q	31742	5	31.6	3	19.9	3	27	complement activation
cornichon homolog 4	16424	1	14.4	1	14.4	1	14.4	intracellular signal transduction
Creatine kinases	47406	15	55.2	18	55.6	13	47.2	catalyzes the transfer of phosphate
Cyclin-dependent kinase 9	43149	2	8.9	6	20.4	2	6.2	cyclin-dependent serine/threonine kinase
Cytochrome b and c	11855	4	43.8	5	26.7	2	22.7	response to mechanical and glucose stimulus
DDRGK domain-containing protein 1	35589	3	15.6	3	15.6	1	4.1	positive regulation of NF-kappaB
Derlin-1 & 2	28896	2	7.6	1	4.4	1	4.4	protease binding
Dimethyladenosine transferase 1	39860	1	4	1	4	1	4	probable dimethyladenosine transferase
DnaJ homolog subfamily B members 6,8,11	40774	2	7.5	5	16.2	2	7.8	HSP binding
Dolichol-phosphate mannosyltransferase 1 & 3	29673	2	14.6	2	23.9	7	16.7	mannosyltransferase
<u>Dynein heavy & light chain 1 & 2</u>	10530	1	37.1	3	64	1	33.7	intracellular motility

STUB1 & TRIM56	35290	1	4	1	2.6	1	0.5	polyubiquitin precursors
ELAV1	36240	5	20.6			3	15.6	RNA binding
Elongation factor 1	50451	2	4.1	2	17.4	6	20.3	protein metabolism
ELOVL5	32755	2	9.3	1	4.3	1	4.3	fatty acid elongase
EMC	34982	5	21.9	3	14.2	4	17.5	ER membrane complex subunits 2,3,6,10
Endothelial differentiation-related factor 1	16359	1	10.1	1	10.1	1	10.1	endothelial cell differentiation
Enhancer of rudimentary homolog	12422	2	36.5	4	44.2	1	16.3	osteoblast differentiation
**EPCAM**	35594	1	5.1	1	5.1	1	5.1	cell-cell adhesion molecule
Erlin-2	38044	4	18	8	33.3	2	10	lipid rafts, critical in IP3 signaling
Eukaryotic initiation factor 4A-I,II,III	46353	21	60.1	21	60.8	22	60.8	cellular protein metabolic processes
Eukaryotic translation elongation factor 1-6	19855	1	5.7	5	33.3	27	62.5	guanine nucleotide exchange
EXOSC	21780	3	29.7	6	43.1	6	28.3	Exosome complex components
F-actin-capping protein	33157	1	8.7	1	3.5	3	14	blocks barbed ends of F-actin filaments
FHL1	38006	8	33.4	8	33.4	8	33.4	Four and a half LIM domains protein 1
General transcription factor IIH & II-I	1E+05	4	5.2	6	6.6	3	3.1	contributes to protein kinase activity
GLULP3	42665	3	13.9	3	13.1	1	4.3	Glutamine synthetase
Glutathione S-transferases	23569	8	48.6	3	31.4	1	7.6	cytoplasmic glutathione S-transferases
GAPDH	36201	8	38.2	3	13.7	5	29	catalyzes carbohydrate metabolism
GTP-binding protein 10 & Rheb & SAR1a	43191	1	2.1	5	20.4	2	5.9	GTPase activity
Guanine nucleotide-binding protein beta-2	35511	19	79.2	19	79.2	19	79.2	SH2 domain binding
HNRNPA1	38837	11	36.8	16	36.2	11	45	Heterogeneous nuclear ribonucleoproteins
HSP10	10925	9	80.4	6	62.7	1	25.5	chaperone binding
Huntingtin-interacting protein K	14656	1	11.6	1	11.6	1	11.6	chaperone like protein
Immediate early response 3-interacting protein 1	9020	2	34.1	2	34.1	2	34.1	may play a role in ER stress response
Interferon-induced transmembrane protein 1	14126	1	12.8	1	12.8	1	12.8	receptor signaling protein activity
Interleukin enhancer-binding factor 2	43263	14	56.2	16	60.5	15	51.3	protein binding
Keratin, type I & II	66170	10	18.6	8	15.8	13	25.3	cytoskeletal proteins
Keratinocyte-associated protein 2	14840	1	12.5	1	12.5	1	12.5	oligosaccharyltransferase
Kinectin	2E+05	1	2.5	2	2.9	1	2.5	involved in intracellular organelle motility
LAMTOR1 & 2 & 3	17848	5	38.5	1	8.1	1	8.1	late endosomal/lysosomal adaptor, MAPK & MTOR activator
Lectin, mannose-binding 2	40545	6	20.2	5	21.9	4	17.7	high mannose glycoprotein binding, facilitates sorting & trafficking
Leucine-rich repeat-containing protein 59 & 7	35308	6	26.7	9	39.7	10	45	facilitates transport of FGF1 through nuclear pores
LLP homolog	15329	1	23.3	3	29.5	2	23.3	long-term synaptic facilitation
Lupus La protein	46979	2	6.9	1	3.4	6	25.2	protein binding
Magnesium transporter protein 1	38411	3	9	3	9	1	5.7	may have a role in N-glycosylation
Mago nashi homolog	17210	3	18.5	3	32.2	2	14.4	RNA splicing
Malectin	32385	4	15.4	4	15.4	3	13	regulates glycosylation
Membrane magnesium transporter 1	14734	1	18.3	1	22.9	1	18.3	ion transmembrane transporter
Membrane-associated progesterone receptor 1 & 2	21772	10	47.7	7	43.6	2	21.5	progesterone steroid receptor
Metadherin	63856	2	6.9	3	9.6	1	2.2	targets of C-MYC transcriptional activation
TRM112	14304	2	21.6	4	37.6	2	21.6	protein methyltransferase activity
Minor histocompatibility antigen H13	41747	1	3.2			1	5.8	aspartic endopeptidase, intramembrane cleaving
Mitochondrial import receptor TOM22 & 5	15512	2	16.2	2	16.2	1	8.5	transmembrane transporter
mRNA turnover protein 4	27657	1	6.3	6	37.2	2	8.8	unknown
Myosin 6B, 12A, 15	19839	3	22.8	5	34.5	5	40.4	hexameric ATPase cellular motor protein

N-alpha-acetyltransferase 10 & 50	26613	1	8.9	9	66.3	1	4.3	acetyl group transfer
Nascent polypeptide-associated subunit alpha	2E+05	6	3.8	7	3.9	6	3.8	associates with BTF3 to form NAC
NEDD3	40802	1	3.3	9	39.2	6	21.3	Developmentally-regulated GTP-binding protein
NipSnap 1 & 2	33460	5	23.6	4	12.9	3	13	may be involved in vesicular transport
Nuclease-sensitive element-binding protein 1	35903	7	43.8	8	48.8	5	27.5	obesity linked gene ?
Nucleolin	76625	9	15.6	6	11.8	5	8.9	angiogenesis ?
Nucleophosmin	32726	2	7.8	5	22.4	4	25.9	several processes including regulation of ARF/p53 pathway
Nucleoside diphosphate kinase A & B	20703	8	61.5	9	61.5	9	56.7	metastasis inhibition factor nm23
Nucleoside-triphosphatase,cancer-related	20928	1	6.3	4	26.3	1	6.3	dephosphorylation
Oligosaccharyltransferase OSTC	16932	1	14.1	1	14.1	2	14.1	protein N-linked glycosylation via asparagine
PCMT1	24792	3	20.7	1	12.3	1	12.3	L-isoaspartate(D-aspartate) O-methyltransferase
Peptidyl-prolyl cis-trans isomerases	18229	10	66.7	9	47.2	5	38.2	catalyze isomerization, cyclosporin binding
Peroxiredoxin-1, 2, 5, 6	22324	9	39.7	6	35.7	6	35.7	antioxidants, reduce hydrogen peroxide & alkyl hydroperoxides
PHD finger-like domain-containing 5A	13138	3	32.7	3	47.3	4	40.9	subunit of splicing factor 3b
phosphatase 2A & PGAM5	32213	7	26.6	5	22.3	2	10.6	Phosphatases
Poly(rC)-binding protein 1 & 2	37987	5	19.4	5	25.2	4	15.9	multifunctional
POTE ankyrin domain family member E	1E+05	7	10.1	7	10.8	8	10.2	proapoptotic protein
PRA1 family protein 2 & 3	21600	2	20.2	1	6.2	1	10.1	L-glutamate and protein transport
Pre-mRNA-splicing factor SPF27	26229	1	5.8	3	20.9	3	20	mRNA splicing
Profilin-1 & 2	15216	6	54.3	3	30	1	20	small actin-binding proteins
Programmed cell death protein 5 & 6	21912	4	22.5	2	12.6	1	5.8	upregulated during apoptosis
Proliferation-associated protein 2G4	44101	11	33.8	17	44.9	17	54.8	interacts with ErbB3, transduces growth regulatory signals
Proteolipid protein 2	17022	2	18.4	2	18.4	2	18.4	may function as an ion channel
Pyrroline-5-carboxylate reductase 1 & 2	33568	4	16	5	25.9	5	25.4	conversion of pyrroline-5-carboxylate to proline
Ran	24579	3	20.8	2	16.2	2	11.1	formation/organization of the microtubule network
SEC22b	24806	4	25.6	4	25.6	3	20.9	complex with SNARE, ER-Golgi protein trafficking
Sec31B	1E+05	2	1.1	2	1.7	2	1.1	unknown
Sec61 alpha & beta	10025	2	26	2	26	2	37.5	secretory & membrane polypeptides insertion into ER
Serine/arginine-rich splicing factors	27842	9	39.9	6	34.8	6	40.9	activates or represses splicing
Serpin H1	46525	1	6.2			2	6.2	serine proteinase inhibitors, cancer marker
SERPINE1	44995	6	23	5	22.1	7	30.6	inhibitor of tissue plasminogen activator & urokinase
Signal peptidase complex 1&2&3	25272	1	26.5	7	33.2	5	28.8	peptidase
Signal recognition particle 14&19&9	16374	5	44.4	5	44.4	6	57	cellular metabolic processes
Single-stranded DNA-binding protein	17249	5	48	3	20.3	3	27.7	involved in mitochondrial biogenesis
Small nuclear ribonucleoprotein E,F,SmD1,D2,D3	13632	8	55.9	3	31.7	9	66.9	RNA and protein binding
small nuclear ribonucleoprotein G-like protein 15	8595	4	36.8	4	36.8	4	47.4	not found
Small nuclear ribonucleoprotein-associated protein B	24765	7	24.6	7	24.6	6	18.8	involved in pre-mRNA splicing
Splicing factor 3B subunit 5&6, U2AF	14690	6	52	5	44.8	5	59.2	RNA splicing
SRA stem-loop-interacting RNA-binding protein	12398	2	22	3	34.9	1	22	negative regulation of mitochondrial RNA catabolism
Stonin-2	1E+05	1	1	1	1	1	1	clathrin-associated sorting proteins, internalization
Stromal cell-derived factor 2-like protein 1	23812	2	16.7	3	25.8	1	19.9	chaperone binding
Surfeit locus protein 4	30602	4	18.6	1	3.7	2	13.8	protein binding
TBC1 domain family member 10B	87658	1	0.9	1	0.9	1	0.9	RAB family, intracellular vesicle trafficking
Thioredoxin-dependent peroxide reductase	28017	5	25	7	41	6	29.3	unknown
TMEM emp24 domain proteins	25131	6	32.4	6	29.2	5	22.8	vesicular protein trafficking

TMEM205	21469	4	27	3	27	2	27	possibly drug resistance
TMEM230	13180	1	9.2	1	9.2	1	9.2	unknown
Trans-2,3-enoyl-CoA reductase	36410	4	13	2	7.1	1	3.2	elongation of fatty acid chains, last step
Translocon-associated protein subunit α, δ, γ	19158	4	30.6	4	30.6	4	21	protein translocation across the ER membrane
Trifunctional enzyme subunit beta	51547	1	2.3			1	2.3	catalyzes mitochondrial beta-oxidation of long chain fatty acids
Tubulin alpha-1B,beta,beta4b	50804	10	34.4			4	16.4	structural constituent of cytoskeleton
Vacuolar ATPase assembly protein VMA21	11347	3	34.7	2	24.8	2	24.8	chaperone for assembly of lysosomal vacuolar ATPase
VAMP3	11359	3	40	3	40	2	40	docking/fusion of synaptic vesicles with presynaptic membrane
Vesicle-associated membrane protein VAPA	28103	5	30.1	6	41.4	4	24.9	vesicle trafficking, membrane fusion, complex assembly, cell motility
Vesicle-associated membrane protein VAPB	27439	5	27.2	7	28	2	16.9	interact with VAMP1 & VAMP2, vesicle trafficking
Voltage-dependent anion-selective channel proteins	30868	7	36.7	7	36.7	2	7.4	transmembrane electron transport
WD repeat-containing protein 61 & 82	33731	2	12.1	2	12.8	2	12.1	transcriptional regulation
Zinc finger CCHC domain-cont. protein 3 & 560	44389	2	7.2	7	23	4	12.6	zinc ion binding

		HEK-E-C		-E(mAG)-C		
Protein Description	Da	hits	%Cov	hits	%Cov	family / function
3-beta-hydroxysteroid-Delta(8),Delta(7)-isomerase	26564	1	9.6	1	9.6	integral membrane protein of the ER
3-hydroxyacyl-CoA dehydratase 2 & 3	43360	5	17.7	6	17.7	Fatty acid biosynthesis
7-dehydrocholesterol reductase	55195	1	4.6	1	4.6	catalyzes 7-dehydrocholesterol to cholesterol conversion
Acylphosphatase-1	11254	2	23.2	1	10.1	hydrolysis of carboxyl-phosphate bonds
ADP/ATP translocase	33059	5	17.8	5	17.8	mitochondrial carrier subfamily
ATP synthase subunits	11421	2	24.3	2	23.3	transmembrane transporter activity
Beta-2-microglobulin	13820	1	8.4	1	8.4	component of the MHC class I complex
C10orf35	13230	2	22.3	3	27.3	interacts with Solute carrier family 39, member 11
Calcyclin-binding protein	26308	1	3.1	1	3.9	calcium-dependent ubiquitination, proteosomal degradation
Carbonic anhydrase 2	29285	8	49.6	2	18.1	reversible hydration of carbon dioxide
Cardiotrophin-like cytokine factor 1	25388	1	3.6	1	3.6	activates the Jak-STAT signaling cascade
**CD81**	26476	2	18.2	1	8.5	tetraspanin
**CD9**	25969	1	4.4	1	4.4	tetraspanin
CDGSH iron-sulfur domain-containing protein	12362	2	25.9	3	34.3	regulation of oxidation
PIS1	23865	1	5.2	1	5.2	biosynthesis of phosphatidylinositol
Cytosolic 5'-nucleotidase 3A	38266	1	1.8	1	3.9	dephosphorylation of nucleoside 5'-monophosphates
D-dopachrome decarboxylase-like protein	14414	1	15.7	1	9.7	lyase activity
DJ-1	20050	4	33.9	1	13.8	chaperone, protects against oxidative stress & cell death
Emerin	29033	1	8.7	4	29.1	mediates membrane anchorage to the cytoskeleton
ES1 protein homolog	28495	1	12.3	1	3	unknown
HSD17B12	34416	3	11.9	1	4.8	estradiol 17-beta-dehydrogenase activity
FAM207A	25441	1	5.7	3	21.3	unknown
FAM3C	24950	2	10.6	1	4.8	cytokine activity
Fructose-bisphosphate aldolase A	39851	6	24.5	1	3.8	glycolysis, conversion of fructose-1,6-bisphosphate
FXYD6	10706	1	23.2	1	1.2	encodes phosphohippolin, affects Na,K-ATPase
Glutaredoxin-related protein 5	16732	1	8.9	1	8.9	normal iron homeostasis
high mobility group protein B1-like	24394	2	12.8	1	5.7	protein binding
Inhibitor of IkB	39399	1	3.4	1	3.4	novel p53 target gene, proapoptotic function

jagunal homolog 1	21111	1	6.6	1	6.6	transmembrane protein, early secretory pathway
L-lactate dehydrogenase A & B	36900	9	26.3	3	11.1	catalyzes L-lactate & NAD to pyruvate & NADH
Lysocardiolipin acyltransferase 1	49344	1	2.4	1	3.1	phospholipid metabolic process
Lysophospholipid acyltransferase 5	56511	1	1.8	1	1.8	phospholipid metabolic process
Macrophage migration inhibitory factor	12639	2	17.4	1	7.8	additional role in integrin signaling
Malate dehydrogenase	35937	12	48.8	2	8.3	pivotal role in the malate-aspartate shuttle
Methyltransferase-like protein 7A	28814	1	6.1	4	27.1	methylation
Mitochondrial import TIM14 & 16 & 50	12491	1	12.1	1	12.1	mitochondrial import inner membrane translocase
Myotrophin	13058	2	25.4	2	25.4	NFkappa B p50-p65 conversion to homodimers
NADH dehydrogenases	30337	2	9.8	2	22.6	binds STAT3, can function as tumor suppressor
Phosphatidylethanolamine-binding protein 1	21158	4	35.8	2	17.1	Raf kinase inhibitory protein
Polyadenylate-binding protein 2	32843	1	3.6	1	3.6	crucial regulator of VEGF mRNA
Prefoldin	14202	1	17.2	1	11.7	chaperone, binds/stabilizes newly synthesized polypeptides
Prohibitin 1 & 2	33276	8	39.1	2	7.7	Antiproliferative activity
Proliferating cell nuclear antigen	29092	3	23.8	3	28.7	cofactor of DNA polymerase delta
Rab-11A	24492	1	6	0	7.4	secretory pathways, protein transport
Rab-14	24110	2	12.1	2	12.1	intracellular membrane trafficking
Rab-1B	22328	6	38.8	1	12.9	early secretory pathway, vesicle transport
Rab-6A				2	10.6	regulates trafficking in retrogradeand an anterograde directions
Rab-7a	23760	4	28.5	2	9.7	vesicle traffic late endosomes & late endosomes to lysosomes
Ran	23467	2	10.9	1	5.5	formation & organization of microtubule network
Rap-1A	21316	2	17.9	1	6.5	regulates signaling pathways (proliferation, adhesion, malignancy)
Replication protein A 14	13674	1	8.3	1	8.3	protein binding
RhoA	22096	1	8.8	1	8.8	Rho protein signal transduction
SEC11A	20612	3	16.2	2	11.2	peptidase S26B family (migration, invasion, metastasis)
Na+/K+ transporting ATPase subunit beta-3	31834	4	17.9	2	10.4	Na/K electrochemical gradients across plasma membrane
Sphingolipid delta(4)-desaturase DES1	38012	1	5	1	5	membrane fatty acid desaturase family
Thioredoxin-like protein 4A	16889	1	7.7	2	16.9	protein binding, splicing
Titin	4E+06	0	0.1	5	27.6	multiple functions
TMEM109	26194	2	9.1	1	4.9	stress response
TMEM33	28302	2	8.5	1	4.9	unknown
TMEM43	44904	0	2.5	3	12	protein binding and self association
TMEM97	21005	1	4.5	1	4.5	controls cellular cholesterol levels
Triosephosphate isomerase	31057	10	47.6	2	10.1	catalyzes isomerization of G3P and DHAP in glycolysis & gluconeogenesis
Triple QxxK/R motif-containing protein	9677	1	9.3	2	9.3	unknown
Tubulin-specific chaperone A	12904	3	27.8	1	4.4	capturing & stabilizing beta-tubulin intermediates
Up-reg. during skeletal muscle growth protein5	6510	1	25.9	1	25.9	unknown
VAMP8	11488	4	32	2	24	SNARE family, synaptic vesicle fusion with presynaptic membranes
Vitamin K epoxide reductase complex 1	18622	1	8	1	5.7	reducing vitamin K 2,3-epoxide to enzymatically activated form
yippee-like 5	14061	2	21.5	1	21.5	interacts with RanBPM and RanBP10 (cell cycle)

		HEK-E-C		
Protein Description	Da	hits	%Cov.	family / function
Acetyl-CoA acetyltransferase	45456	1	3.0	reversible formation of acetoacetyl-CoA from acetyl-CoA

Actin-related protein 2/3 complex	34426	1	3.7	control of actin polymerization
ADP-ribosylation factor	20741	5	37.0	activation of Na(+)-selective current
BAX	21285	1	5.7	heterodimer with BCL2, apoptotic activator
B-cell receptor-associated protein 31	28031	3	14.2	transport from ER to Golgi, caspase 8-mediated apoptosis
C14orf1	15968	2	13.6	ergosterol biosynthetic protein 28
myeloid-derived growth factor	18897	2	13.9	unknown function
**CD147**	42573	6	21.6	EMMPRIN, basigin; Ig superfamily, important in tumor progression
CD54	48935	1	5.7	intercellular adhesion molecule 1
CD59	14795	1	9.4	inhibitor of complement membrane attack complex
Citrate synthase	51908	2	5.8	synthesis of citrate from oxaloacetate and acetyl coA
coagulation factor deficiency protein 2	16494	1	11.6	complex with lectin mannose binding protein, coagulation factors V and VIII transport
CXADR	40575	1	6.3	Coxsackievirus and adenovirus receptor
Cytoskeleton-associated protein 2	77509	1	1.8	stabalizes microtubules, regulation of cell division
Delta-CoA isomerase	36136	3	11.3	beta-oxidation of unsaturated fatty acids
Desmoglein-2	1E+05	1	1.5	expressed in colon, -carcinoma, simple stratified epithelia, found in desmosomes
Electron transfer flavoprotein	35400	4	17.4	shuttles electrons between flavoprotein dehydrogenases and flavoprotein ubiquinone oxidoreductase
Endoplasmic reticulum protein 29	29032	1	4.6	processing of secretory proteins within the ER
ER-Golgi intermediate protein 1	32971	1	7.9	cycling membrane protein, increase turnover of other proteins
Enoyl-CoA hydratase	31823	1	7.2	mitochondrial fatty acid beta-oxidation pathway
Flotillin-2	47434	2	6.8	caveolae-associated, thought to function in neuronal signaling
GLOD4	35170	1	4.2	Glyoxalase domain-containing protein 4
Glycosyltransferases	50940	6	17.8	tightly associated subunit of oligosaccharyltransferase
G-rich sequence factor 1	53606	2	4.6	stimulate translation of viral mRNAs in vitro
HPRT1	24792	3	15.1	conversion of hypoxanthine to inosine and of guanine to guanosine monophosphate
HSP beta-1	22826	2	21	stress resistance and actin organization
Inositol 1,4,5-trisphosphate receptor1	3E+05	1	0.5	intracellular receptor for inositol 1,4,5-trisphosphate, mediates calcium release
Isocitrate dehydrogenase	51333	1	6.2	catalyze the oxidative decarboxylation of isocitrate to 2-oxoglutarate
KBTBD3	70775	1	1.2	Kelch repeat and BTB domain-containing protein 3
Lactoylglutathione lyase	20992	1	4.3	the catalysis and formation of S-lactoyl-glutathione from methylglyoxal condensation and reduced glutatione
Mannose-6-phosphate receptor	31487	1	8.7	transport of mannose-6-phosphate-containing acid hydrolases from the Golgi complex to lysosomes
MOB kinase activator 1A	25235	1	5.6	metal ion and protein binding
NAD(P)H-hydrate epimerase	31996	1	4.2	apolipoprotein A-I-binding protein
Non-specific lipid-transfer protein	59640	2	5.5	intracellular lipid transfer protein
Nucleolysin TIA-1 p40	43278	1	7.8	major granule-associated species, 15-kDa protein
Phosphatidylinositol-bind. clathrin assembly protein	70881	1	2.1	AP2-dependent clathrin-mediated endocytosis
Phosphoglycerate kinase 1	44985	9	32.4	conversion of 1,3-diphosphoglycerate to 3-phosphoglycerate
Phosphoglycerate mutase 1	28900	5	25.2	catalyzes 3-PGA to 2-PGA in glycolysis
phosphotyrosine phosphatase	18487	1	7	concentrated in focal adhesions
Prostaglandin E synthase 3	18971	1	18.8	chaperone cofactor-dependent protein refolding
Proteasome subunit alpha-1,4,6,7,& beta-1,3,4	29822	2	10.3	catalytic subunits
Protein Phosphatase 2A	28682	2	10.8	PolyA and protein binding
Rab-18	23248	3	25.2	membrane trafficking in organelles and transport vesicles
Rab-2A	23702	2	12.7	protein transport from ER to Golgi
Rab-35	23296	3	21.9	Interplay with Arf6, cargo recycling, coordinates adhesion and migration.
Rab-5C	23696	2	18.1	ensure fidelity in docking / fusion of vesicles with acceptor compartment

Ral-A	23723	1	7.3	increases paracellular permeability, decreases incorporation into TJs
Rap-2c	20959	2	12	activate transcriptional activity of serum response element
<u>Ras GTPase-activating-like protein IQGAP2</u>	2E+05	1	0.4	interacts with cytoskeleton, cell adhesion molecules, signaling molecules, regulates morphology & motility
RER1	23057	2	14.8	retention of ER membrane proteins in the ER
Reticulocalbin-2	36911	1	6.6	similarity to a high affinity Ca(+2)-binding motif
<u>Rho GDP-dissociation inhibitor 1</u>	23250	1	7.4	GDIs slow dissociation rate of GDP from membrane-bound rabs
Rho guanine nucleotide exchange factor 40	2E+05	1	0.4	similar to guanosine nucleotide exchange factors for Rho GTPases
<u>Secretory carrier-associated membrane protein 2</u>	37082	1	6.1	carriers to the cell surface in post-golgi recycling
SEP15	18236	1	9.9	may have redox function
SET-binding protein	2E+05	1	1	bind the SET nuclear oncogene (involved in DNA replication)
SETSIP	34861	3	12.3	endothelial cell differentiation
SH3 domain-binding glutamic acid-rich-like protein	12766	1	10.5	signal transduction
Sideroflexin-1	35881	3	14.6	ion transmembrane transporter activity
SLC16A1	54593	1	2.4	proton-linked monocarboxylate transporter
<u>Small VCP/p97-interacting protein</u>	8551	1	14.3	Small VCP/p97-interacting protein
Sorcin	21947	2	16.7	regulates intracellular calcium homeostasis
<u>Spartin</u>	73358	1	4.2	regulates endosomal trafficking
<u>Stathmin</u>	17292	1	16.8	regulates microtubule filament system, destabilizes microtubules
Stomatin-like protein 2	38624	1	4.2	decreased survival in gastric Ca
Succinyl-CoA ligase subunit alpha	36626	1	9	catalyzes succinyl CoA and ADP or GDP to succinate and ATP or GTP
SUMO2	10921	1	12.6	nuclear transport, transcriptional regulation, apoptosis, protein stability
<u>Synaptophysin-like protein 1</u>	28889	1	4.2	synaptic transmission, vesicles
<u>Syntaxin-12</u>	31736	2	15.2	SNARE binding
<u>Syntaxin-7</u>	29911	3	14.6	SNARE interactions in vesicular transport
Thioredoxin domain-containing protein 12&5	19365	2	14	catalyzes disulfide bond formation, similar to disulfide isomerases
Transaldolase	37688	2	10.1	nonoxidative pentose phosphate pathway for nucleic acid synthesis and lipid biosynthesis
Transgelin-2	22548	4	27.6	thought to be a tumor suppressor
<u>Translocating chain-associated membrane protein 1</u>	43215	1	2.9	influences glycosylation, translocation of secretory proteins
Tub	55845	1	1.2	transcription regulator, translocates to the nucleus in response to phosphoinositide hydrolysis
Ubiquitin-conjugating enzyme E2 N	17184	3	30.3	DNA postreplication repair
<u>VAMP7</u>	25261	2	14.1	SNARE family, late endosomes and lysosomes, fusion of transport vesicles to target membranes

MALDI-TOF analysis was performed with the lower part of a 10% SDS gel of cld7 coimmunoprecipitating molecules. All proteins revealing at least one protein-specific peptide as well as the percent of the protein covered by the isolated peptide(s) are shown. The molecular weight of the protein is included. As far as closely related variants or submembers of a protein were recovered, these have been mentioned, but mW and number of hits are only presented for one family member that showed the highest number of hits. presented as one entity. The main function of the molecules or their classification into protein families are indicated. Coimmunoprecipitating molecules are presented in alphabetic order and separated into proteins coimmunoprecipitating with HEK-EpC-cld7 independent of the EpC mAG mutation (blue) and of cld7 palmitoylation (red), or only independent of cld7 palmitoylation (no red) or dependent on the EpC-cld7 association and cld7 palmitoylation (only black).
Proteins, whose association with cld7 was confirmed by coimmunoprecipitation are printed in bold. Proteins engaged in vesicle formation or traffic are underlined.
