# Supplementary material for: The importance of claudin-7 palmitoylation on membrane subdomain localization and metastasis-promoting activities
Source: Cell Commun Signal. 2015 Jun 9;13:29. doi: 10.1186/s12964-015-0105-y (PMC4459675; doi:10.1186/s12964-015-0105-y)
Supplement: Additional file 4: — The impact of cld7 palmitoylation on cld7 phosphorylation, presenilin2 and β-catenin expression. [file 12964_2015_105_MOESM4_ESM.pdf]

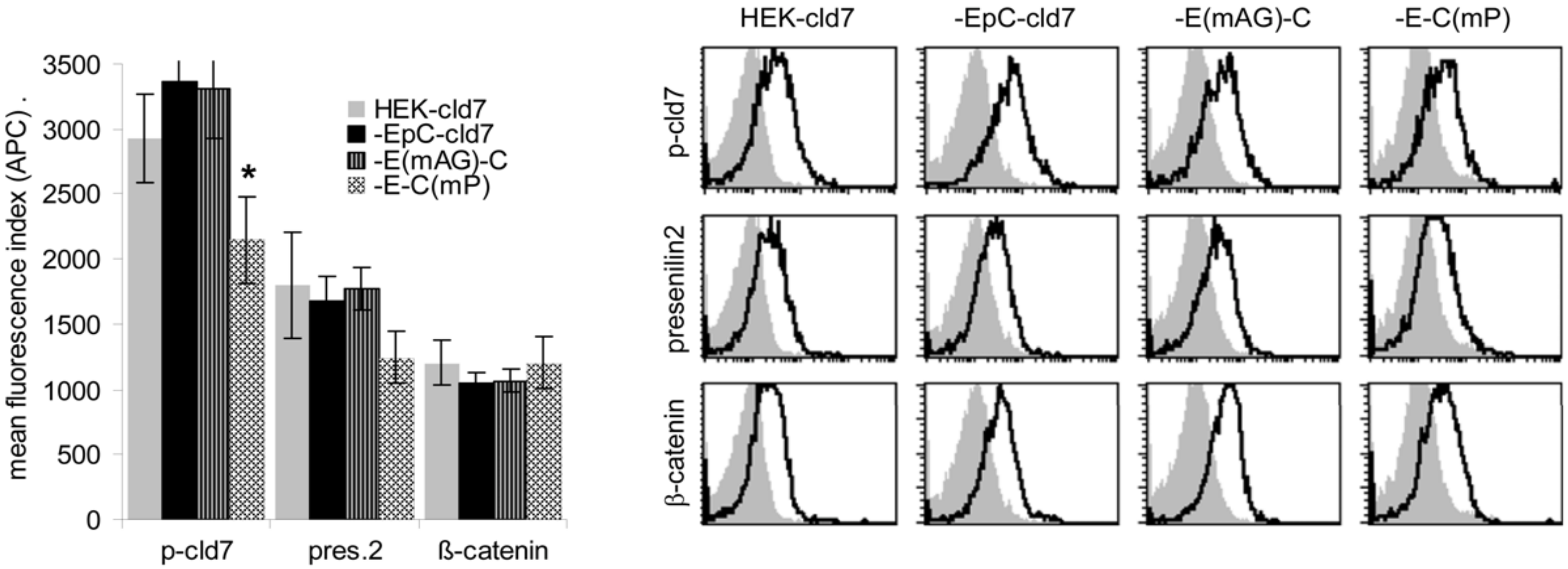

Add.File 4

Additional File 4. The impact of cld7 palmitoylation on cld7 phosphorylation, presenilin2 and β-catenin expression Flow cytometry of p-cld7, presenilin2 and β-catenin in PMA-stimulated transfected HEK cells; the mean fluorescence index (% stained cells x mean intensity of staining ± SD; three assays) and representative examples are shown; significant differences between HEK-cld7 versus HEK-EpC-cld7, HEK-EpC^{mAG}-cld7 and HEK-EpC-cld7^{mPalm} cells: * Palmitoylated cld7 has a slight impact on cld7 phosphorylation.
