# Supplementary material for: The importance of claudin-7 palmitoylation on membrane subdomain localization and metastasis-promoting activities
Source: Cell Commun Signal. 2015 Jun 9;13:29. doi: 10.1186/s12964-015-0105-y (PMC4459675; doi:10.1186/s12964-015-0105-y)
Supplement: Additional file 5: — Antibodies and chemicals. [file 12964_2015_105_MOESM5_ESM.pdf]

Additional File 5

5A Chemicals

substance	dose	supplier
AnnexinV-FITC / -APC	variable	Becton Dickinson, HD, G
Cisplatin	1-30 µg / ml	Sigma, Munich, G
Methyl-β-cyclodextrin	20 mM, 30 min	Merck, Darmstadt, G
Matrigel	invasion: 1:5	Becton Dickinson, HD, G
Phalloidin	0.5 µg / ml	Becton Dickinson, HD, G
PI	variable	Becton Dickinson, HD, G
PMA	10^{-8} M	Sigma Munich, G
TACE inhibitor (TAPI)	50 µm	Calbiochem, Darmstadt, G
Palmitoyl. inhibitor (2-BP)	15 µM	Sigma, Munich, G

5B Antibodies

Antibody	origin	supplier
Actin	mouse	Becton Dickinson, HD, G
β-catenin	rabbit	Becton Dickinson, HD, G
casein kinaseβ	mouse	Becton Dickinson, HD, G
CD29	mouse	Becton Dickinson, HD, G
CD49e	mouse	Becton Dickinson, HD, G
CD49f	mouse	Becton Dickinson, HD, G
CD104	rabbit	Becton Dickinson, HD, G
CD133	rabbit	Becton Dickinson, HD, G
CD147	mouse	Becton Dickinson, HD, G
CD166	mouse	Becton Dickinson, HD, G
cld3	rabbit	Santa Cruz, HD, G
cld4	guinea pig	Santa Cruz, HD, G
cld5	rabbit	Santa Cruz, HD, G
cld7	guinea pig	ref [2]
E-cadherin	mouse	Becton Dickinson, HD, G
EGFR	rabbit	AnaSpec, San Jose, Ca, US
EpCAM (D5.7)	mouse	ref [3]
EpIC	rabbit	home made (no reference)
ezrin	rabbit	Sigma, Munich, G
FAK	rabbit	Becton Dickinson, HD, G
FGF	rabbit	Becton Dickinson, HD, G
FN	mouse	Becton Dickinson, HD, G
MMP2	rabbit	Dianova, Hamburg, G
MMP3	rabbit	Santa Cruz, HD, G
MMP7	rabbit	Santa Cruz, HD, G
MMP9	rabbit	Dianova, Hamburg, G
MMP13	rabbit	Dianova, Hamburg, G
MMP14	rabbit	Santa Cruz, HD, G
N-Cadherin	mouse	Becton Dickinson, HD, G
Notch	mouse	Biolegend, San Diego, Ca, US
Oct3/4	rabbit	Santa Cruz, HD, G
paxillin	rabbit	Becton Dickinson, HD, G

5B continued

Antibody	origin	supplier
p-β-catenin	rabbit	BioTrend, Cologne, G
p-casein kinaseβ	rabbit	Abcam, Cambridge, UK
p-cld7	rabbit	Sigma, Munich, G
p-ezrin	rabbit	Santa Cruz, HD, G
p-FAK	rabbit	Cell Signaling, G
phosphotyrosine	mouse	Becton Dickinson, HD, G
presenilin2	rabbit	Santa Cruz, HD, G
p-src	rabbit	Cell Signaling, G
slug	rabbit	Santa Cruz, HD, G
snail	rabbit	Santa Cruz, HD, G
src	rabbit	Santa Cruz, HD, G
SOX2	rabbit	Santa Cruz, HD, G
SSEA4		Becton Dickinson, HD, G
TACE	rabbit	Santa Cruz, HD, G
TGFβ	mouse	Becton Dickinson, HD, G
TNFα	hamster	Becton Dickinson, HD, G
tubulin	mouse	Becton Dickinson, HD, G
twist	rabbit	Becton Dickinson, HD, G
vimentin	mouse	Becton Dickinson, HD, G
vinculin	goat	Santa Cruz, Heidelberg, G
ZEB-1	rabbit	Santa Cruz, Heidelberg, G
ZO-1	rabbit	Santa Cruz, HD, G
dye or biotin labeled secondary antibodies / Streptavidin		Dianova, Becton Dickinson, Amersham

References

1. Kuhn S, Koch M, Nübel T, Ladwein M, Antolovic D, Klingbeil P, Hildebrand D, Moldenhauer G, Langbein L, Franke WW, Weitz J, Zöller M. A complex of EpCAM, claudin-7, CD44 variant isoforms, and tetraspanins promotes colorectal cancer progression. Mol Cancer Res. 2007;5:553-67.
2. Ladwein M, Pape UF, Schmidt DS, Schnölzer M, Fiedler S, Langbein L, Franke WW, Moldenhauer G, Zöller M. The cell-cell adhesion molecule EpCAM interacts directly with the tight junction protein claudin-7. Exp Cell Res. 2005;309:345-57.
3. Matzku S, Wenzel A, Liu S, Zöller M. Antigenic differences between metastatic and nonmetastatic BSp73 rat tumor variants characterized by monoclonal antibodies. Cancer Res. 1989;49:1294-9.
